# The Systematic Landscape of Nectin Family and Nectin-Like Molecules: Functions and Prognostic Value in Low Grade Glioma

**DOI:** 10.3389/fgene.2021.718717

**Published:** 2021-12-01

**Authors:** Yunhe Han, Cunyi Zou, Chen Zhu, Tianqi Liu, Shuai Shen, Peng Cheng, Wen Cheng, Anhua Wu

**Affiliations:** Department of Neurosurgery, The First Hospital of China Medical University, Shenyang, China

**Keywords:** low-grade glioma (LGG), Nectin, nectin-like molecules, prognosis, immune microenvironment

## Abstract

**Objective:** Nectin and nectin-like molecules (Necls) are molecules that are involved in cell–cell adhesion and other vital cellular processes**.** This study aimed to determine the expression and prognostic value of nectin and Necls in low grade glioma (LGG).

**Materials and Methods:** Differentially expressed nectin and Necls in LGG samples and the relationship of nectin family and Necls expression with prognosis, clinicopathological parameters, and survival were explored using The Cancer Genome Atlas (TCGA), the Chinese Glioma Genome Atlas (CGGA), and Repository of Molecular Brain Neoplasia Data (REMBRANDT) databases. Univariate and multivariate Cox analysis models were performed to construct the prognosis-related gene signature. Kaplan-Meier curves and time-dependent receiver operating characteristic (ROC) curves and multivariate Cox regression analysis, were utilized to evaluate the prognostic capacity of the four-gene signature. Gene ontology (GO)enrichment analysis and Gene Set Enrichment Analyses (GSEA) were performed to further understand the underlying molecular mechanisms. The Tumor Immune Estimation Resource (TIMER) was used to explore the relationship between the four-gene signature and tumor immune infiltration.

**Results:** Several nectin and Necls were differentially expressed in LGG. Kaplan–Meier survival analyses and Univariate Cox regression showed patients with high expression of *NECTIN2* and *PVR* and low expression of *CADM2* and *NECTIN1* had worse prognosis among TCGA, CGGA, and REMBRANDT database. Then, a novel four-gene signature was built for LGG prognosis prediction. ROC curves, KM survival analyses, and multivariate COX regression indicated the new signature was an independent prognostic indicator for overall survival. Finally, GSEA and GO enrichment analyses revealed that immune-related pathways participate in the molecular mechanisms. The risk score had a strong negative correlation with tumor purity and data of TIMER showed different immune cell proportions (macrophage and myeloid dendritic cell) between high- and low-risk groups. Additionally, signature scores were positively related to multiple immune-related biomarkers (IL 2, IL8 and IFNγ).

**Conclusion:** Our results offer an extensive analysis of nectin and Necls levels and a four-gene model for prognostic prediction in LGG, providing insights for further investigation of CADM2, NECTIN1/2, and PVR as potential clinical and immune targets in LGG.

## Introduction

Low grade glioma (LGG) is a prevalent type of primary malignancy of the central nervous system, with high heterogeneity in biological behavior. (Ostrom et al., 2013; Zeng et al., 2018). Despite treatment involving maximum surgical resection combined with postoperative radiotherapy and chemotherapy, most LGG cases finally progress to therapy-resistant high-grade aggressive glioma, and the prognosis is not improved considerably (Claus et al., 2015). Thus, identifying novel prognostic factors for LGG is extremely important. Several biomarkers, such as the isocitrate dehydrogenase (*IDH*) mutation and co-deletion of chromosome arms 1p and 19q (1p/19q codeletion) have been integrated into the 2016 WHO brain tumor classification for further subclassification. (Hartmann et al., 2010; Louis et al., 2016). However, these biomarkers cannot sufficiently provide risk stratification of LGG patients, especially for diseases with a variant genetic background. Thus, using multiple advanced molecular platforms to identify novel prognostic biomarkers to improve the stratification of LGG is urgent and of great importance.

As the main component in the innate immune system, NK cells detect and eliminate damaged cells through immune surveillance, which plays an important role in tumor immunity, such as glioma. (Vivier et al., 2011). For example, infiltration of NKp44^+^ NK cells is present in many types of gliomas and positively correlates with the expression of pro-inflammatory NK cell cytokine genes, such as IFNG and TNF. (Barrow et al., 2018). PDGF-DD-activated NK cells infiltration and KLR family receptors expression on NK cells were positively related with a more favorable prognosis in LGG patients. (Sun et al., 2021). Moreover, there was a higher infiltration of NK cells in IDH1 mutant glioma patients, and this was correlated with a better prognosis. (Ren et al., 2019). However, due to the immune escape mechanism of glioma, NK cells are limited in number and function in gliomas. The number of CD3^−^CD56^+^ NK cells decreased in glioma patients, and these NK cells highly expressed TIM3, an immune checkpoint related to glioma progression (Han et al., 2014), leading to a decreased capability of IFN-γ secretion. (Li et al., 2018). In addition to abnormal NK cells, gliomas themselves express molecules that promote immune escape. The carbohydrate-binding protein galectin-1 was overexpressed by both mouse and rat malignant glioma, which suppressed NK immune surveillance. (Baker et al., 2014). Galectin-1 suppression, leading to the recruitment of Gr-1^+^/CD11b^+^ myeloid cells and NK1.1+NK cells into the brain tumor microenvironment, culminating in tumor clearance. (Baker et al., 2016). These results mean that targeting to restore the function of NK cells in the microenvironment may be more conducive to the treatment of glioma.

In addition to immune surveillance, NK cells can also maintain self-tolerance, and these different functions are performed by a wide spectrum of NK cells. These cells are programmed with diverse capacities to interact via surface receptors with major histocompatibility class I molecules (MHC-I), and this process is called education. (Boudreau and Hsu, 2018a). In humans, NK education generally occurs between MHC-I and inhibitory receptors, such as the CD94/NKG2A heterodimer, or killer immunoglobulin-like receptors (KIR). (Boudreau and Hsu, 2018b). Educated NK cells target cells and tissue lacking “self” MHC-I expression. (Ljunggren and Kärre, 1985). Tumor cells down-regulate human leukocyte antigen (HLA) expression, which means that “missing-self” is then recognized and lysed by NK cells. (Martinet and Smyth, 2015; Sun et al., 2015). However, recent studies have shown that HLA expression is maintained at some detectable level and/or can be easily induced in most tumors, a conclusion supported by the successful observation of immune checkpoint inhibitors in the treatment of many different cancers. (Garrido et al., 2016; Malmberg et al., 2017). This means that, in addition to the interaction between MHC-I molecules and related inhibition receptors, NK cells also have other inhibitory molecules which combine with non-MHC-I molecules to perform immune surveillance functions. (He et al., 2017; He and Tian, 2017). Therefore, the study of these non-MHC-I binding inhibitory receptors on NK cells will provide new insights into tumor immune escape and immunotherapy.

Nectin and nectin-like molecules (Necls) are cell adhesion molecules that are involved in cell–cell adhesion and vital cellular activities (Takai et al., 2003; Takai and Nakanishi, 2003). Cumulative evidence has revealed that nectin and Necls play an important role in tumorigenesis and tumor immunosurveillance. Immunoglobulin receptors on natural killer (NK) and CD8^+^ T cells can recognize nectin and Necl proteins in various types of cancer (Chan et al., 2012). Furthermore, NECTIN2 participates in tumorigenesis and can inhibit CD8^+^ T-cell functions (26). Moreover, PVR, the ligands of DNAM-1-activating NK receptor, presents a high level of expression in glioblastoma stem cells. (Castriconi et al., 2009) NECTIN4 mRNA and protein expression are upregulated in hepatocellular carcinoma (Ma et al., 2016). As the major entry molecule, NECTIN one plays an important role in oncolytic herpes simplex virus-1 (oHSV) entering glioma. (Friedman et al., 2009; Friedman et al., 2012; Friedman et al., 2018; Alayo et al., 2020). Besides, NECTIN1 and CADM3 inhibit the migration and invasion of glioblastoma cell lines. (Gao et al., 2009; Yin et al., 2010). However, systematic analyses of nectin and Necl expression profiles and their function in LGG are still lacking.

The present study comprehensively assessed the expression profile and prognostic value of the nectin family and Necl members based on updated public resources and integrative bioinformatics analysis. Thereafter, we constructed a nectin family- and Necl-based prognostic model using LGG cases from three different cohorts, with more accurate predictive ability, by combining the nectin family and Necl members and clinical characteristics. Considering the vital role of nectin and Necls in tumor immunology, the relationship between this signature and the LGG immune microenvironment was further explored.

## Materials and Methods

### ONCOMINE Database

The expression level of individual NECTIN family members and Necls in different types of cancer were determined by analysis based on the ONCOMINE database (AnnArbon, MI, United States) (http://www.oncomine.org/) (Rhodes et al., 2004). Oncomine provided 715 dataset microarray information and some online functions. Student’s t-test was used to get the *p*-value for the difference of NECTIN family genes between cancer specimens and normal controls. The threshold of fold change and *p*-value were set at 2 and 0.05 respectively.

### LGG Expression Data for This Study

The expression level and clinical data of LGG patients were gathered from the genomic data commons The *Cancer* Genome Atlas (TCGA) LGG cohort (downloaded from http://xena.ucsc.edu/) (Goldman et al., 2020), the Chinese Glioma Genome Atlas (CGGA; http://www.cgga.org.cn) databases, and REMBRANDT database. The TCGA LGG cohort contained 463 tumor samples, the CGGA cohort contained 172 tumor samples, and the REMBRANDT cohort contained 107 tumor samples. The basic characteristics of the LGG patients used in research are shown in Table 1.

**TABLE 1:** Basic characteristics of LGG patients.

		TCGA (463)			CGGA (172)			REMBRANDT (86)		
		High risk	Low risk	*p*-value	High risk	Low risk	*p*-value	High risk	Low risk	*p*-value
Age				0.015			0.005			0.357
(years)	<55	170	192		73	80		14	51	
	≥55	62	39		13	6		10	11	
				0.059			0.072			
Gender	male	134	124		57	49		NA		
	female	98	107		29	37				
				0.801			0.591			0.245
Grade	II	79	140		35	64		2	19	
	III	153	91		51	22		22	43	
							0.132			
IDH status	wildtype	81	6		34	10		16	23	
	mutant	151	225		52	76		8	39	
				0.374			0.403			
1p19q status	codel	29	125		9	48		NA		
	non-codel	203	106		77	38				

### The mRNA Expression and Correlation of the NECTIN and Necls in LGG

The comparison of the NECTIN and necls gene expression in different grade LGG tissues and the expression difference of the NECTIN family based on IDH status or 1p19q codeletion status was analyzed by Sanger box tools, a free online platform for data analysis (http://www.sangerbox.com/tool). We utilized the corrplot package to further explore the correlation between NECTIN and necls expression in LGG, the results were visualized using Sanger box tools.

### UALCAN Database

UALCAN website provides a comprehensive assessment of cancer transcriptome data (https://ualcan.path.uab.edu/). (Chandrashekar et al., 2017) The expression level of NECTIN and necl genes in different histology subtype LGG tissues were analyzed by the UALCAN database.

### cBioportal Data Extraction

The cBioportal database provides comprehensive analyses of complex transcriptional and clinical data from The *Cancer* Genome Atlas (TCGA) (https://www.cbioportal.org/). (Cerami et al., 2012) The genomic alterations of the NECTIN family and necl genes alterations (amplification, deep deletion, and missense mutations) in LGG patients were assessed by cBioportal for TCGA.

### Survival Analysis of NECTIN Family and Necls in LGG

The prognostic values of the NECTIN members and necls in the TCGA LGG cohort, CGGA LGG cohort, and REMBRANDT cohort were evaluated using the following two steps: (Ostrom et al., 2013): the patients were divided into two groups based on the half-cut point gene expression by survival R package. Kaplan-Meier method and log-rank test were applied to appraise the influence of the expression of each NECTIN member and necls on the overall survival (OS) of LGG patients in three independent cohorts (Zeng et al., 2018). Univariate Cox proportional hazard regression analysis was used to assess the NECTIN family and necls significantly associated with OS in three databases. The *p*-value of genes both less than 0.05 in (Ostrom et al., 2013) and (Zeng et al., 2018) among three databases were identified as prognosis-related genes for further analysis. All the analyses were performed using the survival package in R 3.6.3 software.

### Signature Construction and Statistical Analysis

A risk formula of the aforesaid survival-related genes was determined by a linear combination of the gene expression levels and weighted with the corresponding regression coefficients from univariate Cox proportional hazard regression analysis. The TCGA cohort was treated as a training set, while the CGGA AND REMBRANDT cohort was the validation set. Patients were classified into high-risk and low-risk groups according to the risk score. The difference of LGG patients’ histological grade, IDH status, and 1p19q codeletion status between the high-risk score group and low-risk score group were presented with *t*-test. The Kaplan-Meier survival curve was used to assess the OS scores between the high- and low-risk groups, and the difference in OS between the two groups was detected by log-rank test, and the receiver operating characteristic (ROC) curve analysis within 0.5, 1, and 3 years was applied to assess the prognostic significance of the risk score model by R packages “survivalROC”. *p* < 0.05 was set as a significant difference in all statistical methods, and the results figures were produced by Sanger box online tools.

### Enrichment Analysis, GSEA and PPI Analysis for Function and Interaction of NECTIN Family and Necls Signature

Pearson correlation analysis was used to obtain genes for functional annotation. Then the top 1,000 genes correlated with the risk score and *p*-value<0.05 were uploaded to the metascape website for Enrichment analysis of Gene Ontology (GO) annotation and visualization (http://metascape.org) (Zhou et al., 2019). We performed Gene Set Enrichment Analysis (GSEA, version 4.0.1; http://software.broadinstitute.org/gsea/index.jsp) to find pathways related to the differential risk score expression in the TCGA LGG tissues. (Subramanian et al., 2005). The annotated gene set file c5. go.bp.v7.4. entrez.gmt (from the Msig database) was used as the reference. A random combination number of 1,000 permutations and a *p*-value<0.05 was set for screening the target pathway. The protein–protein interaction (PPI) network of the genes involved in the risk score formula was performed by Gene Multiple Association Network Integration Algorithm (GeneMANIA; https://www.genem ania. org/) (Warde-Farley et al., 2010).

### Immune Infiltrate Analysis

The difference of immune cell abundance, including B cells, CD4^+^ T cells, CD8^+^ T cells, neutrophils, macrophages, and dendritic cells, between the high and low risk score groups in the TCGA and CGGA cohorts were gained or calculated from tumor immune estimation resource (TIMER; https://cistrome. shinyapps. io/timer/) (Li et al., 2017; Li et al., 2020). Based on expression data, the immune score, stromal score, and tumor purity of each LGG patient were analyzed by “estimate” package to evaluate the levels of immune cell infiltration and stromal cells in tumor tissues. Pearson’s correlation coefficient was calculated to evaluate the associations between the expression of signature risk score and the above three scores or IL2, IL8, and IFNγ expression levels.

## Results

### *NECTIN* Family and Necl Gene Expression Patterns in LGG Samples

**FIGURE 1:**
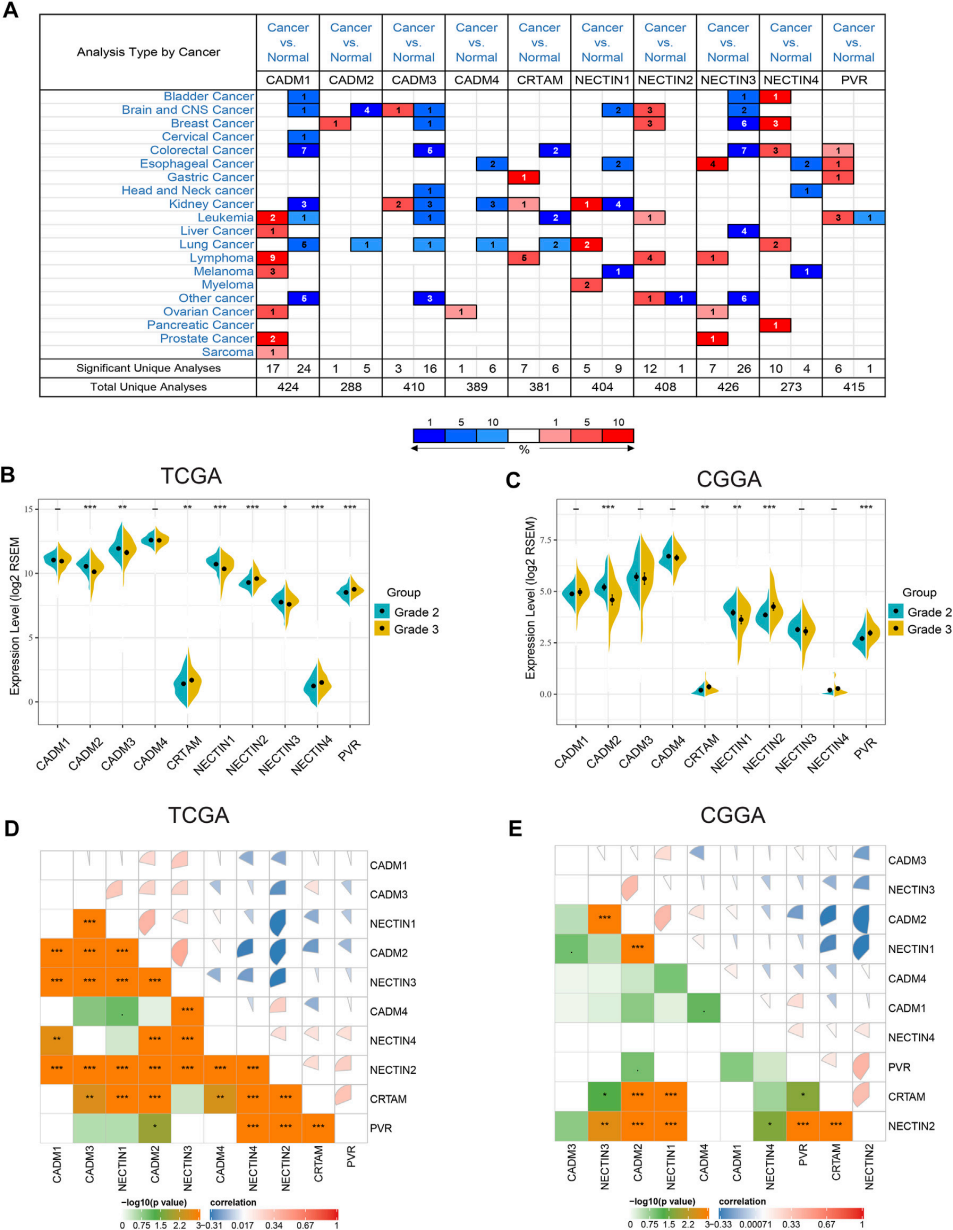
Expression profile of nectin family members and necls in different cancers and LGG **(A)**. The expression level of nectin family members and necls in multiple types of cancers. Red and blue stand for the numbers of datasets with statistically significant (*p* < 0.05) overexpression and under expression levels of nectin family members and necls, respectively. **(B,C)** The expression level of nectin family members and necls were differentially in different grades LGG in TCGAB and CGGA **(C)**. **(D,E)** Correlation plot among nectin family members and necls in TCGA **(D)** and CGGA **(E)**. The color of the pie chart represents the correlation of the expression of two genes (blue: negatively correlated, red: positively correlated). The area of the colored pie is proportional to the strength of mutual correlation. The plot in the lower part represents the Statistical significance (by Spearman test).

First, we refined the chromosome location of all *NECTIN* family and *necl* genes based on a literature review. The details are shown in Table 2. Next, we examined the ONCOMINE database to compare the mRNA expression of the *NECTIN* family between cancer and normal tissues in multiple cancers. As shown in Figure 1A, the expression of *NECTIN* and *necl* genes were different between all types of cancer and corresponding matched normal tissues. *CADM3* and *CADM4* were upregulated in most cancers, whereas *NECTIN2* was downregulated in most cancers. For brain and CNS tumor samples, *CADM1*, *CADM2*, *NECTIN1*, and *NECTIN3* were downregulated, whereas NECTIN2 was overexpressed.

**TABLE 2:** Basic characteristics of NECTIN family genes.

Gene id	Official symbol	Synonym(s)	Exon	Chromosomal location
23,705	CADM1	BL2	13	11q23.3
		ST17		
		IGSF4		
		NECL2		
253,559	CADM2	NECL3	14	3p12.1
		IGSF4D		
57,863	CADM3	BlgR	12	1q23.2
		NECL1		
		TSLL1		
		IGF4B		
199,731	CADM4	NECL4	10	19q13.31
		TSLL2		
		IGF4C		
56,253	CRTAM	CD355	10	11q24.1
5,818	NECTIN1	ED4	10	11q23.3
		PRR		
		CD111		
		PVRL1		
5,819	NECTIN2	HVEB	10	19q13.32
		PRR2		
		CD112		
		PVRL2		
25,945	NECTIN3	PRR3	14	3q13.13
		CD113		
		PVRL3		
81,607	NECTIN4	LNIR	9	1q23.3
		PRR4		
		PVRL4		
5,817	PVR	PVS	8	19q13.31
		HVED		
		CD155		
		NECL5		

Thereafter, we analyzed the relationship between the expression of the *NECTIN* family and *necl* genes and the histological grade of LGG samples in TCGA, CGGA, and REMBRANDT databases, separately. In TCGA samples, the expression levels of *CRTAM*, *NECTIN2*, *NECTIN4*, and *PVR* were upregulated with an increasing grade, whereas *CADM2*, *CADM3*, *NECTIN1*, and *NECTIN3* were downregulated (Figure 1B). In CGGA samples, the expression of *CRTAM*, *NECTIN2*, and *PVR* was upregulated with an increasing grade, whereas *CADM2* and *NECTIN1* were downregulated (Figure 1C). In REMBRANDT samples, the levels of *CADM4*, *NECTIN2*, and *PVR* were upregulated with a more advanced grade, whereas *CADM2* and *NECTIN1* were downregulated (Supplementary Figure S1A). Thus, *NECTIN2* and *PVR* were upregulated, whereas *CADM2* and *NECTIN1* were downregulated in all three databases. Further analysis showed the correlation among *NECTIN* family members in LGG. As shown in Figures 1A,D strong positive correlation existed between *CADM2* and *NECTIN1/3*, a strong negative correlation was found between *NECTIN2* and *CADM2* or *NECTIN1/3* in TCGA LGG samples, and insignificant correlations were identified among nectin family members in CGGA and REMBRANDT databases (Figure 1E, Supplementary Figure S1B).

### Associations Between Gene Alterations of the NECTIN Family and Necls and LGG Subtype, IDH1 Status, and 1P19q Status

**FIGURE 2:**
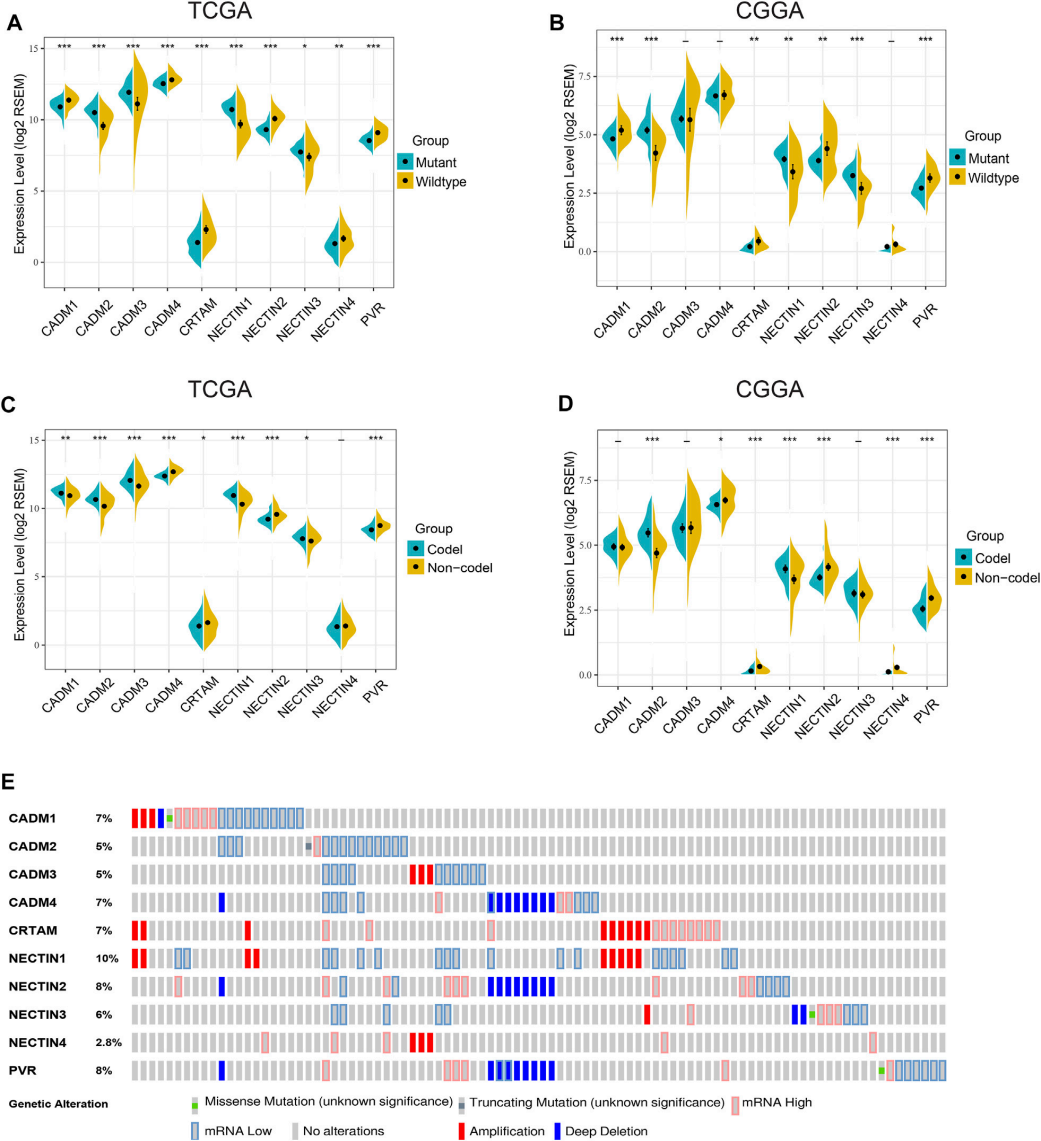
Gene alteration and associations between NECTIN family and necls and LGG molecular subtype. **(A,B)** The expression level of nectin family members and necls were differentially in IDH wildtype and mutant samples in TCGA **(A)** and CGGA **(B)**. **(C,D)** The expression level of nectin family members and necls were differentially in 1p19q codeletion and non-codeletion samples in TCGA **(C)** and CGGA **(D)**. E OncoPrint visual summary of Genomic alterations on query of nectin family members and necls in LGG (cBioportal).

An analysis of 635 LGG samples from the TCGA and CGGA databases independently showed that differential expression of *NECTIN* family and *necl* genes was significantly associated with IDH mutation status, 1p19q codeletion status, and histological subtypes (Figure 2, Supplementary Figure S2). Based on the results of the two databases, *CADM1*, *CRTAM*, *NECTIN2*, and *PVR* were highly expressed in IDH-wild-type samples, whereas *CADM2*, *NECTIN1*, and *NECTIN3* were highly expressed in IDH-mutant samples (Figures 2A,B). Furthermore, *CADM2* and *NECTIN1* were highly expressed in 1p19q-codeletion samples, whereas *CADM4*, *CRTAM*, *NECTIN2*, and *PVR* were highly expressed in 1p19q-non-codeletion samples (Figures 2C,D). An analysis based on the UALCAN website showed the expression differences in the *NECTIN* family and *necls* based on different histological LGG subtypes (Supplementary Figure S2). Next, genetic alterations were analyzed in TCGA LGG patients to obtain in-depth insight into the molecular mechanisms of differential expression of the *NECTIN* family and *necl* genes. As shown in Figure 2E, *NECTIN1* was associated with the highest probability of alterations (10%), followed by *PVR* (8%), whereas *NECTIN4* had the lowest alteration probability (2.8%). These results indicate the close correlation between *NECTIN* family gene expression and a series of significant clinical parameters.

### Identification of Four prognosis‐Related NECTIN Family and Necl Genes in LGG Samples

**FIGURE 3:**
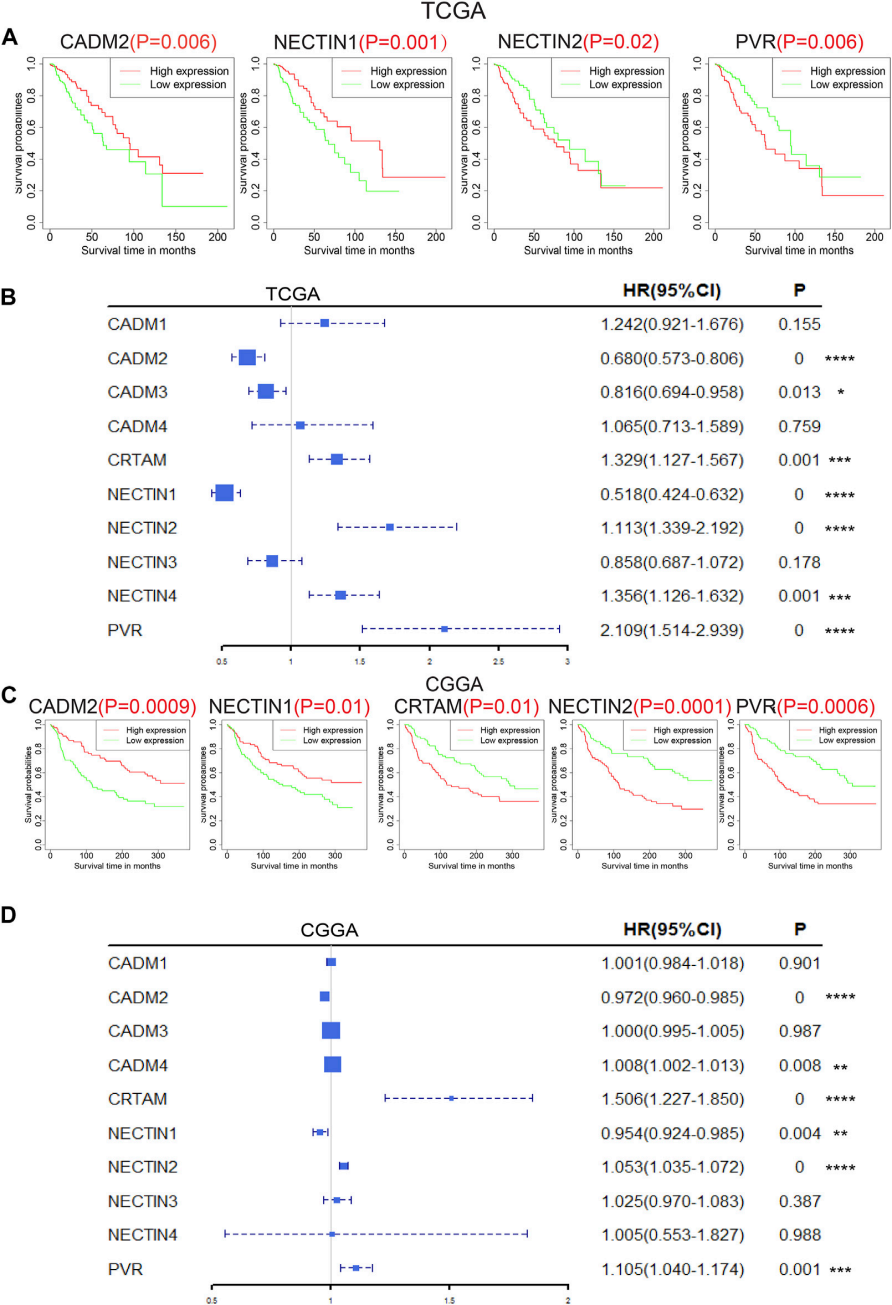
Prognostic value of nectin family members and necls in LGG patients. Survival plot of nectin family members and necls with *p* < 0.05 in TCGA **(A)** and CGGA **(C)**. Forest plot on overall survival of nectin family members and necls with *p* < 0.05 in TCGA **(B)** and CGGA **(D)**.

The prognostic values of *NECTIN* family members and *necls* were analyzed subsequently. The Kaplan–Meier survival curve results of all *NECTIN* family members and *necls* in TCGA, CGGA, and REMBRANDT databases are presented in Figure 3 and Supplementary Figures S3 and S4. Based on an independent analysis of the three databases, patients with a high expression of *NECTIN2* and *PVR* and low expression of *CADM2* and *NECTIN1* had a worse prognosis (*p* < 0.05; Figures 3A,C, Supplementary Figures S4). Following univariate Cox regression analysis results based on the three databases, *CADM2*, *NECTIN1*, *NECTIN2*, and *PVR* were found to exhibit a significant prognostic correlation with overall survival (OS, *p* < 0.05; Figures 3B,D, Supplementary Figure S4). Therefore, we identified the aforementioned four genes as prognosis-related genes of the *NECTIN* family and *necl* in LGG samples.

### Construction of the NECTIN Family- and Necl-Based Signature of LGG Based on TCGA

**FIGURE 4:**
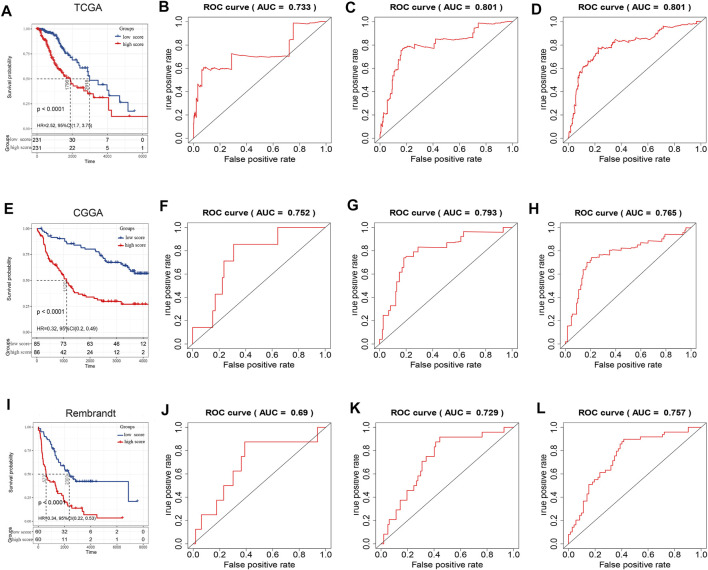
Survival analysis and prognostic performance of risk score in LGG. Kaplan–Meier survival curves based on risk score in TCGA **(A)**, CGGA **(E)**, and Rembrandt cohorts **(I)**. 0.5‐, 1‐, and 3‐years overall survival ROC curves based on risk score in TCGA **(B–D)**, CGGA **(F–H)**, and Rembrandt cohorts **(J–L)**.

After filtering four prognosis-related genes in three databases independently, a risk formula was constructed based on the expression levels of the four genes. The corresponding regression coefficients are as follows: risk score = −0.386 × CADM2 + −0.659 * NECTIN1 + 0.538 ×NECTIN2 + 0.746 × PVR. All LGG patients were divided into high-risk and low-risk groups based on the median risk scores, and Kaplan–Meier survival analysis indicated that the high-risk group had a significantly worse OS than the low-risk group, based on TCGA training (log-rank *p* < 0.0001) and two validation cohorts (CGGA and REMBRANDT, both log-rank *p* < 0.0001; Figures 4A,E,I). Moreover, the risk score model had a better predictive ability of 0.5-, 1-, and 3-years OS, with the area under the ROC curve showing values of 0.733, 0.801, and 0.801 in the TCGA training cohort (Figures 4B–D), 0.752, 0.793, and 0.765 in the CGGA validation cohort (Figures 4F,H), and 0.69, 0.729, and 0.757 in the REMBRANDT validation cohort, respectively, indicating the strong power of the *NECTIN* family-based signature in predicting the OS of LGG patients (Figures 4J–L). Next, we used univariate and multivariate COX analyses based on the clinical characteristics of all patients in the TCGA and CGGA cohorts to verify the independence of the risk score model. The risk score model was significantly correlated with OS, with HR = 1.22 (95% confidence interval [CI] = 1.02–1.46; *p* = 0.03) in the TCGA training cohort, and with HR = 1.50 (95% CI = 1.28–1.84; *p* < 0.001) in the CGGA validation cohort (Table 3).

**TABLE 3:** COX analysis of risk score in TCGA training cohort and CGGA validation cohorts.

Factors	TCGA (training set)				CGGA (validation set)			
	Univariate analysis		Multivariate analysis		Univariate analysis		Multivariate analysis	
	HR (95%CI)	*p*-value	HR (95%CI)	*p*-value	HR (95%CI)	*p*-value	HR (95%CI)	*p*-value
Age	1.059 (1.043–1.075)	<0.001	1.052 (1.035–1.069)	<0.001	1.032 (1.009–1.055)	0.005	1.005 (0.984–1.027)	0.644
WHO Grade	3.173 (2.093–4.811)	<0.001	1.876 (1.202–2.928)	0.006	3.848 (2.500–5.922)	<0.001	3.068 (1.943–4.837)	<0.001
Gender	1.172 (0.805–1.708)	0.408	1.262 (0.849–1.877)	0.25	0.630 (0.415–0.954)	0.029	0.545 (0.355–0.837)	0.006
IDH status	0.166 (0.113–0.246)	<0.001	0.310 (0.181–0.531)	<0.001	0.390 (0.250–0.607)	<0.001	0.909 (0.515–1.605)	0.743
Riskscore	1.660 (1.457–1.892)	<0.001	1.221 (1.020–1.463)	0.03	1.621 (1.394–1.884)	<0.001	1.503 (1.276–1.844)	<0.001

### Related Clinical Characteristics and Pathways Based on Risk Score in LGG Patients

**FIGURE 5:**
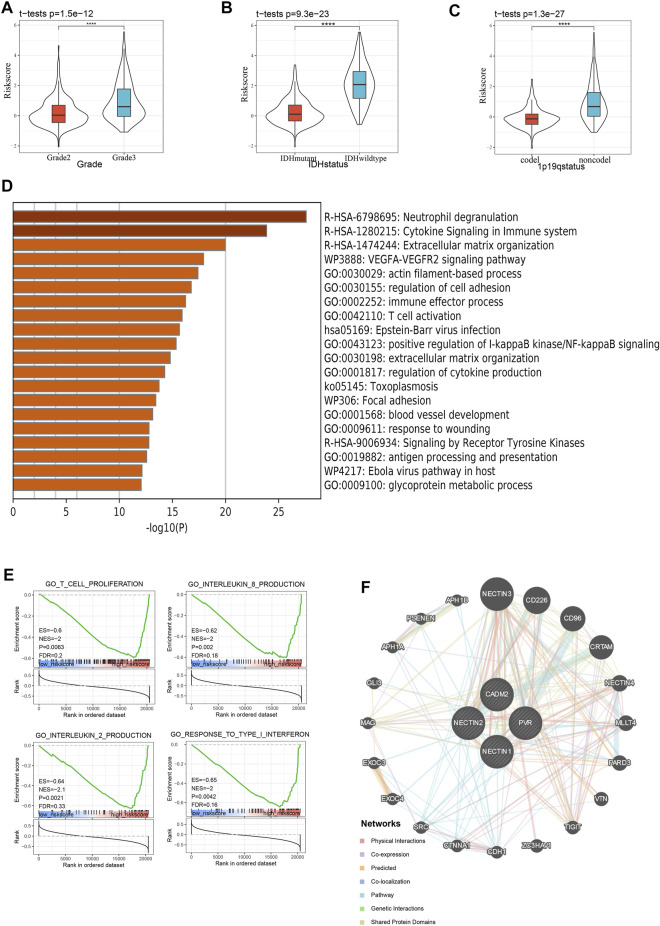
Enrichment and correlation analysis among risk score in TCGA database. **(A-C)** violin plot indicates the relationship between risk score and grade **(A)**, IDH status **(B)**, and 1p19q status **(C)** in TCGA. D Heatmap of top 20 enriched terms of top 1,000 correlated genes with risk score in TCGA LGG samples, colored by *p*-value. E GSEA of risk score in the TCGA LGG cohort indicating an association of risk score with immune response. F PPI network of risk score related genes from GENEMANIA.

First, we explored the distributions of risk scores among LGG cases of different histology grades and observed that the risk score was higher with malignant progression, such as in the high-grade LGG, IDH-wild-type LGG, and 1p19q-non-codeletion LGG samples (Figures 5A–C), which was verified with the CGGA validation cohort (Supplementary Figures S5A–5C). Thereafter, to explore signature-related biological pathways, we first sought to determine the top 1,000 genes correlated with our risk score in TCGA and CGGAThe results are shown in Supplementary Table S1 and Supplementary Table S2. These differential genes were subsequently selected for enrichment analysis in Metascape (http://metascape.org). Interestingly, in addition to the functions mentioned in many studies, such as localization and biological adhesion (Samanta and Almo, 2015), immune system processes such as immune effector process, T cell activation, and cytokine signaling in the immune system were also related to the *NECTIN* family and *necl* genes (Figure 5D, Supplementary Figure S5D). Meanwhile, gene set enrichment analysis (GSEA) revealed that the *NECTIN* family and *necl* signatures were more relevant to some important immune-related pathways and molecules, such as the innate immune response, activation of lymphocytes, IL2, IL8, and interferon (Figure 5E, Supplementary Figure S5E). Moreover, we constructed a protein-protein interaction network (Figure 5F) of signature-related genes with GeneMANIA to explore the potential interactions among them. SRC, an important tyrosine kinase primarily involved in immune regulation, was among the neighboring genes predicted to interact with the four signature genes, further revealing the deep connection between the *NECTIN* family and *necl* signature and immune processes.

### Associations Between NECTIN-Family Signature and Immune Infiltration

**FIGURE 6:**
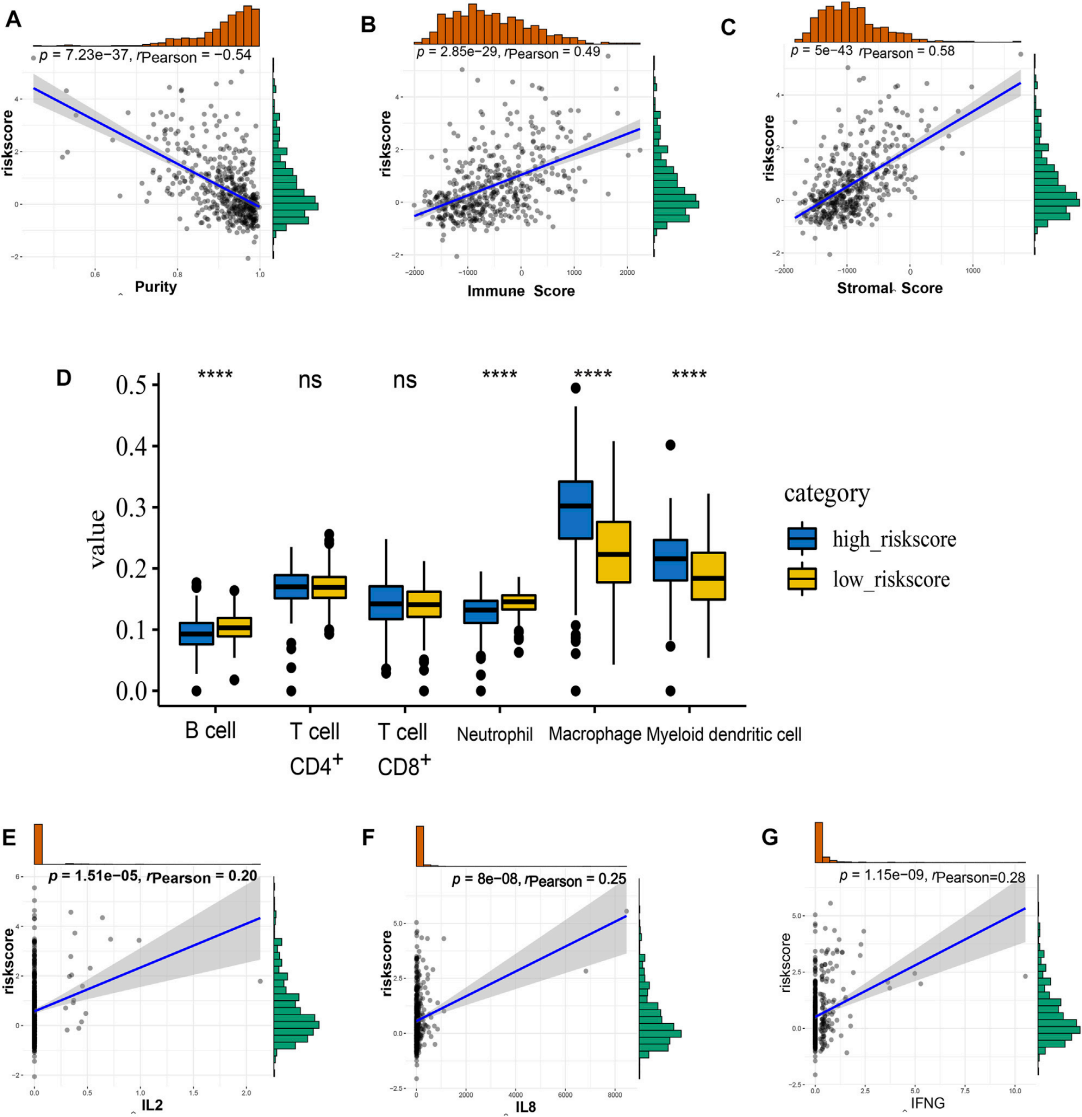
The immune landscape of nectin-based signature in TCGA LGG samples. **(A–C)** The correlation of risk score level with tumor purity **(A)**, immune score **(B)**, and stromal score **(C)**. **(D)** The details of different expression immune cells in high- and low-risk groups i. **(E–G)** The correlation of risk score level with the expression of IL2 **(E)**, IL8 **(F)**, and IFNG **(G)**.

According to the ESTIMATE method, we observed the correlation between risk score and immune, stromal, and tumor purity separately based on TCGA and CGGA cohorts. The risk score was strongly positively correlated with the immune score and stromal score but negatively correlated with tumor purity, indicating that a more complicated immune microenvironment exists in high-risk-score patients (Figures 6A–C, Supplementary Figures S6A–C). Next, TIMER (https://cistrome. shinyapps. io/timer/) was used for each sample in the TCGA cohort to estimate immune cell infiltration. As shown in Figure 6D, the high-risk group had a higher population of macrophages and myeloid dendritic cells. In contrast, the low-risk group had a higher population of B cells and neutrophils. Furthermore, the correlation between the risk score and important immune molecules (Figure 5E) was analyzed by Pearson correlation test, and the results showed that the risk score was positively correlated with IL2, IL8, and IFNγ (Figures 6E–G). These data indicate the important roles of the *NECTIN* family and *necl* signature-related genes in the immune regulation of LGG.

## Discussion

Nectin and necl were initially identified as members of the immunoglobulin (Ig)-like family and participate in calcium-independent cell adhesion (Takai et al., 2003). Disruption of the cell–cell adhesive process has been reported to result in various abnormalities in humans and a series of diseases, including Alzheimer’s disease, reproductive disorders, and tumor metastasis (Mandai et al., 2015; Samanta and Almo, 2015). In addition, nectins and Necls can participate in tumorigenesis through another mechanism. PVR has been implicated in tumor immune regulation. It serves as a potent immune ligand, binding two types of receptors expressed on the cell surfaces of T and NK cells, co-stimulatory immune receptor (CD226), or the inhibitory checkpoint receptors (TIGIT) (O'Donnell et al., 2020). In addition, PVR can also bind to CD96, but the role of CD96 is not clear. On the one hand, in CD96^−/−^ mice displaying hypersensitive NK-cell responses to immune challenge and significant tumor resistance (Blake et al., 2016), blocking CD96^−^CD155 interaction or TGF-β1 restores NK cell immunity against tumors by reversing NK cell exhaustion in liver cancer. (Sun et al., 2019). On the other hand, CD96 promotes NK cell adhesion to target cells expressing PVR, stimulates cytotoxicity of activated NK cells, and has a co-stimulatory role in CD8^+^ T cell activation and effector function (Fuchs et al., 2004; Chiang et al., 2020). The crucial role of the nectin family and Necls in cancers makes them potential biomarkers and therapeutic targets for various types of tumors. NECTIN4 is abnormally highly expressed and contributes to tumor proliferation in pancreatic cancer and hepatocellular carcinoma (Nishiwada et al., 2015; Ma et al., 2016). PVRL2 expression is greater in numerous cancers relative to that in normal tissues, including colorectal carcinoma and breast, endometrial, lung, and ovarian cancers (Karabulut et al., 2016; Whelan et al., 2019). However, gene expression patterns based on the entire nectin family and Necls have not been previously studied in LGG. In the present study, we analyzed the RNA-seq data from TCGA, CGGA, and REMBRANDT databases to identify the four gene expression patterns related to clinical parameters (histological grade, IDH status, and 1p19q non-codeletion status). *NECTIN2* and *PVR* were highly expressed in IDH-wild-type, 1p19q-non-codeletion, and higher histological grade samples, whereas *CADM2* and *NECTIN1* were highly expressed in IDH-mutant, 1p19q-codeletion, and lower histological grade samples. Previously, the role of these four genes in LGG had only been studied rarely.

In this study, univariate Cox proportional hazard regression and the Kaplan–Meier survival analyses of *NECTIN* family genes and Necls in the three databases also provided convincing prognostic evidence. Patients with high expression of *NECTIN2* and *PVR* had a worse prognosis than those with low expression, whereas patients with high expression of *CADM2* and *NECTIN1* had a better prognosis than those with low expression. These results further verified the above conclusion. Patients with IDH1 mutant and 1p19q codeletion exhibit better prognosis than those with IDH1-wild-type and 1p19q non-codeletion gliomas (Hartmann et al., 2010; Hainfellner et al., 2014). The relationship between the expression of four prognostic-related genes (*CADM2*, *NECTIN1*, *NECTIN2*, and *PVR*) and clinical parameters (histological grade, IDH status, and 1p 19q status) was consistent with this theory. Therefore, these prognostic-related genes could serve as promising predictive candidates, offering more evidence for the prediction of survival outcomes for LGG patients with accuracy. Subsequently, we established and validated a risk score model based on the expression of prognosis-related genes (*CADM2*, *NECTIN1*, *NECTIN2*, and *PVR*), which was independently correlated with OS in patients with LGG, as determined via multivariate Cox analysis. Moreover, the risk score model had a favorable predictive ability of 0.5-, 1-, and 3-years survival, as indicated by the ROC curve. Patients with IDH1-mutant, 1p19q-codeletion, and lower histological grade LGG had a lower risk score, which was associated with better OS. Next, to identify the *NECTINs* and *Necls* involved in biological pathways in LGG, the top 1,000 differentially expressed genes with the largest fold-change and a *p*-value < 0.05 between low-risk score and high-risk score groups were used to conduct GO enrichment analyses. Interestingly, these genes were primarily enriched in the immune system and localization and adhesion processes (*p* < 0.01), which was consistent with the role of nectin and Necls in different types of tumors (Raveh et al., 2009; Chan et al., 2012; Chan et al., 2014; Whelan et al., 2019). The results of GSEA also showed a strong relationship between the risk score and several immune pathways and molecules, such as the innate immune response, activation of lymphocytes, IL-2, and IL-8. These provided strong evidence that the dysregulation of risk score-related genes plays an important role in the immune regulation of LGG. There is currently no literature on the association between *CADM2* and tumor immune processes, and thus, our risk score model might suggest a new direction for future research.

Components of the glioma microenvironment confer important genomic, biological, and clinical implications. Tumor purity and nontumor cell components also play a vital role in tumor biology. In glioma, patients with lower tumor purity have more immune cells or stromal cells in their microenvironment and poor prognosis (Zhang et al., 2017). In the present study, our risk score had a strong negative correlation with tumor purity and a positive correlation with the stromal and immune score, which was consistent with our prognosis results. Moreover, our results show that patients with a higher risk score have more macrophage and myeloid dendritic cell infiltration. The functions of glioma-associated macrophages (tumor-associated macrophages) are inhibited and are weak in antigen-presenting and cross-priming capacity (Hussain et al., 2006; Raychaudhuri et al., 2011; Gabrusiewicz et al., 2016). A previous study reported that the dendritic cells in glioma were inhibited by fibrinogen-like protein 2 in glioma stem cells (Yan et al., 2019). These reports further suggest that the *NECTIN* and *Necl* signature might effectively reflect the infiltration of immune cells in the LGG microenvironment.

## Conclusion

Taken together, by assessing the expression profile in three independent databases, we identified four prognosis-related genes among *NECTINs* and *Necls* (*CADM2*, *NECTIN1*, *NECTIN2*, and *PVR*) with great potential to serve as therapeutic targets and prognostic biomarkers in LGG. We discussed the potential pathway participating in the novel four-gene signature and found that the risk score model plays an important role in the immune process of LGG. To address the limitations of the present study, our team will address the biological behavior and molecular mechanism of *NECTINs* and *Necls* in LGG through further experiments, which will improve our understanding and have implications for treating patients with efficacy.

## Data Availability

The original contributions presented in the study are included in the article/Supplementary Material, further inquiries can be directed to the corresponding authors.

## Abbreviations

BP, Biological process; CGGA, The Chinese Glioma Genome Atlas; GO, Gene Ontology; LGG, low grade glioma; Necls, nectin-like molecules; OS, The overall survival rate; PPI, Protein–Protein Interaction; TCGA, The Cancer Genome Atlas.
