# Supplementary figures and images for: The Systematic Landscape of Nectin Family and Nectin-Like Molecules: Functions and Prognostic Value in Low Grade Glioma
Source: Front Genet. 2021 Dec 1;12:718717. doi: 10.3389/fgene.2021.718717 (PMC8672115; doi:10.3389/fgene.2021.718717)

A

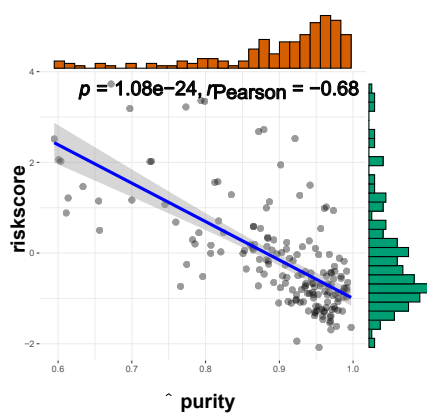

B

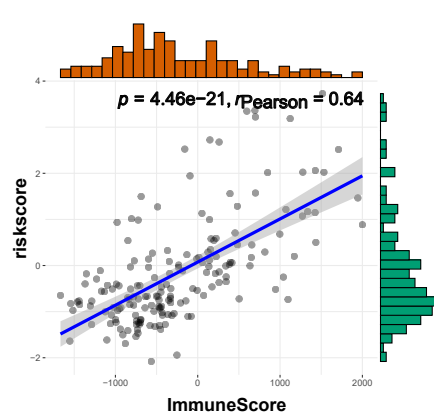

C

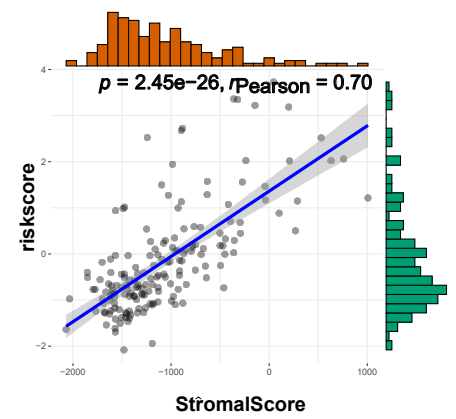

D

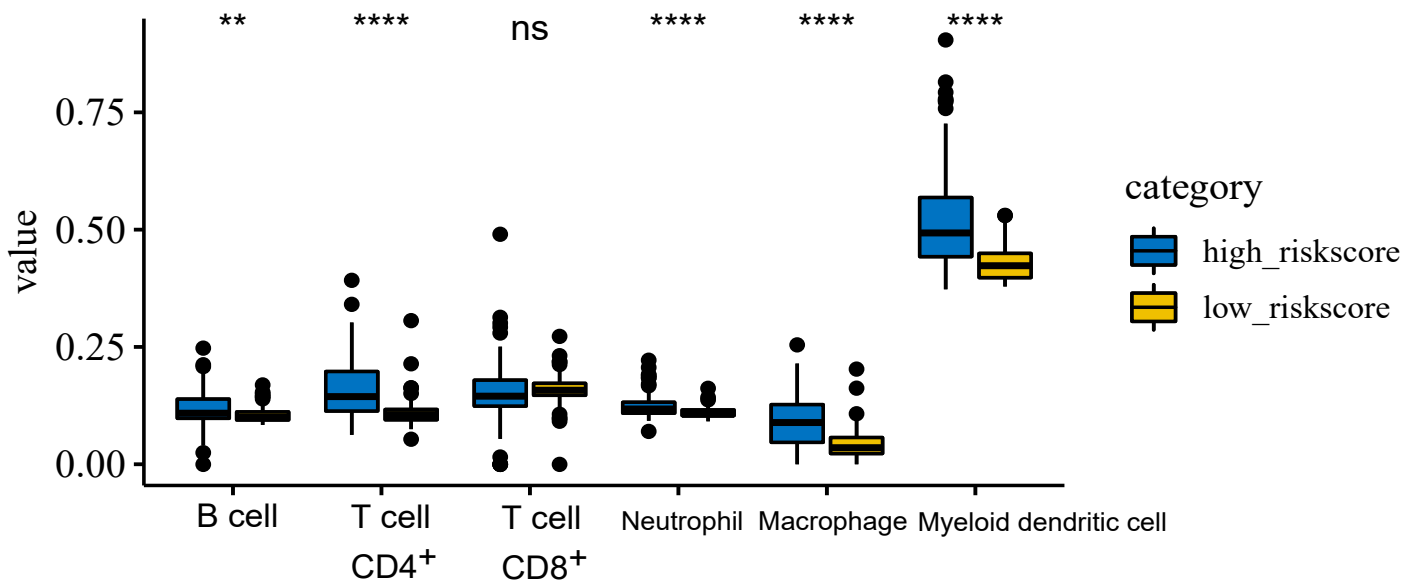

E

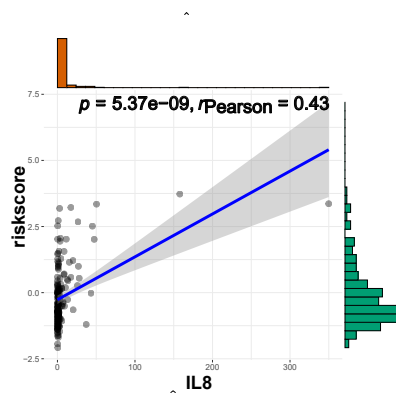

Supplement: Supplementary file 1 [file DataSheet7.PDF]

A

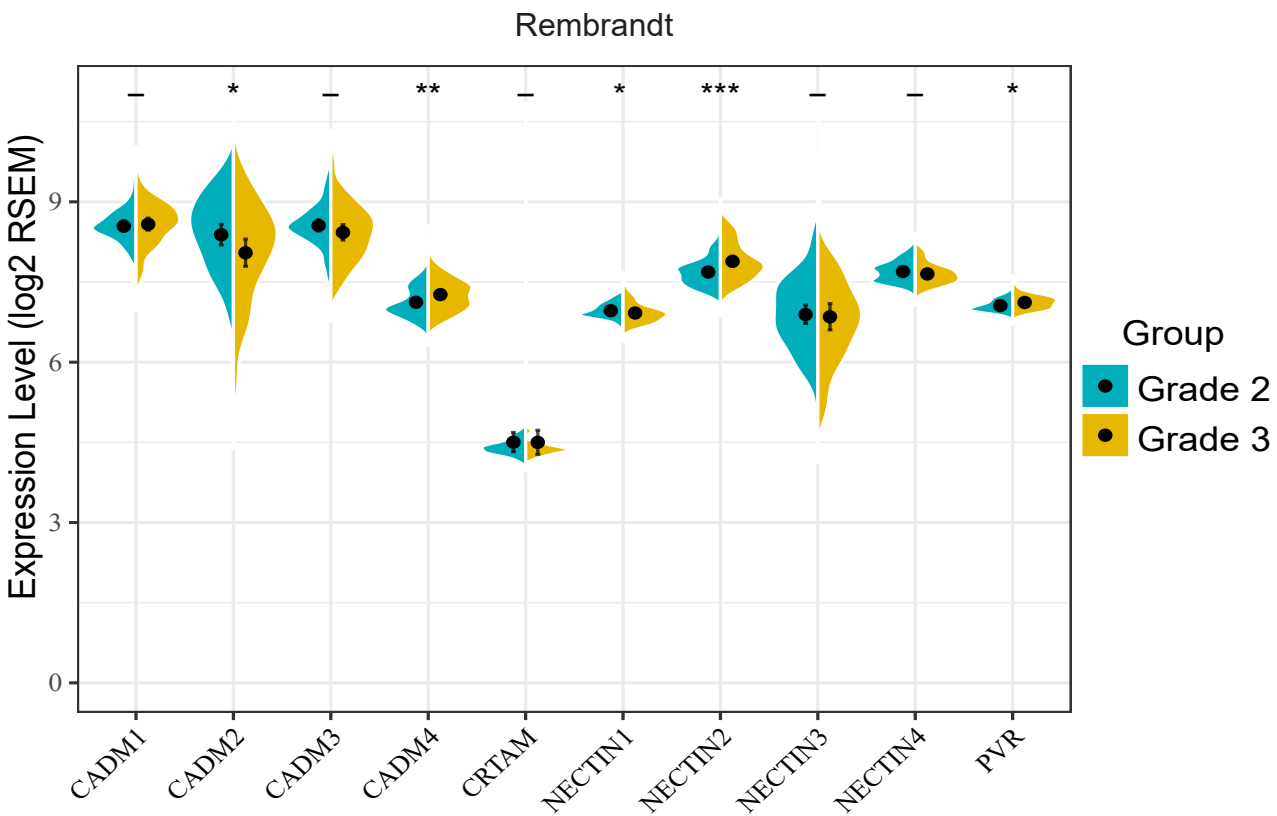

B

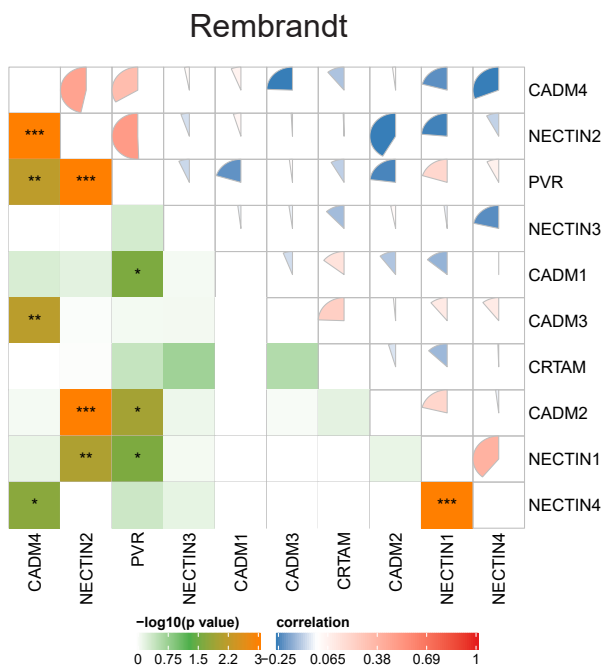

Supplement: Supplementary file 2 [file DataSheet2.PDF]

## A TCGA

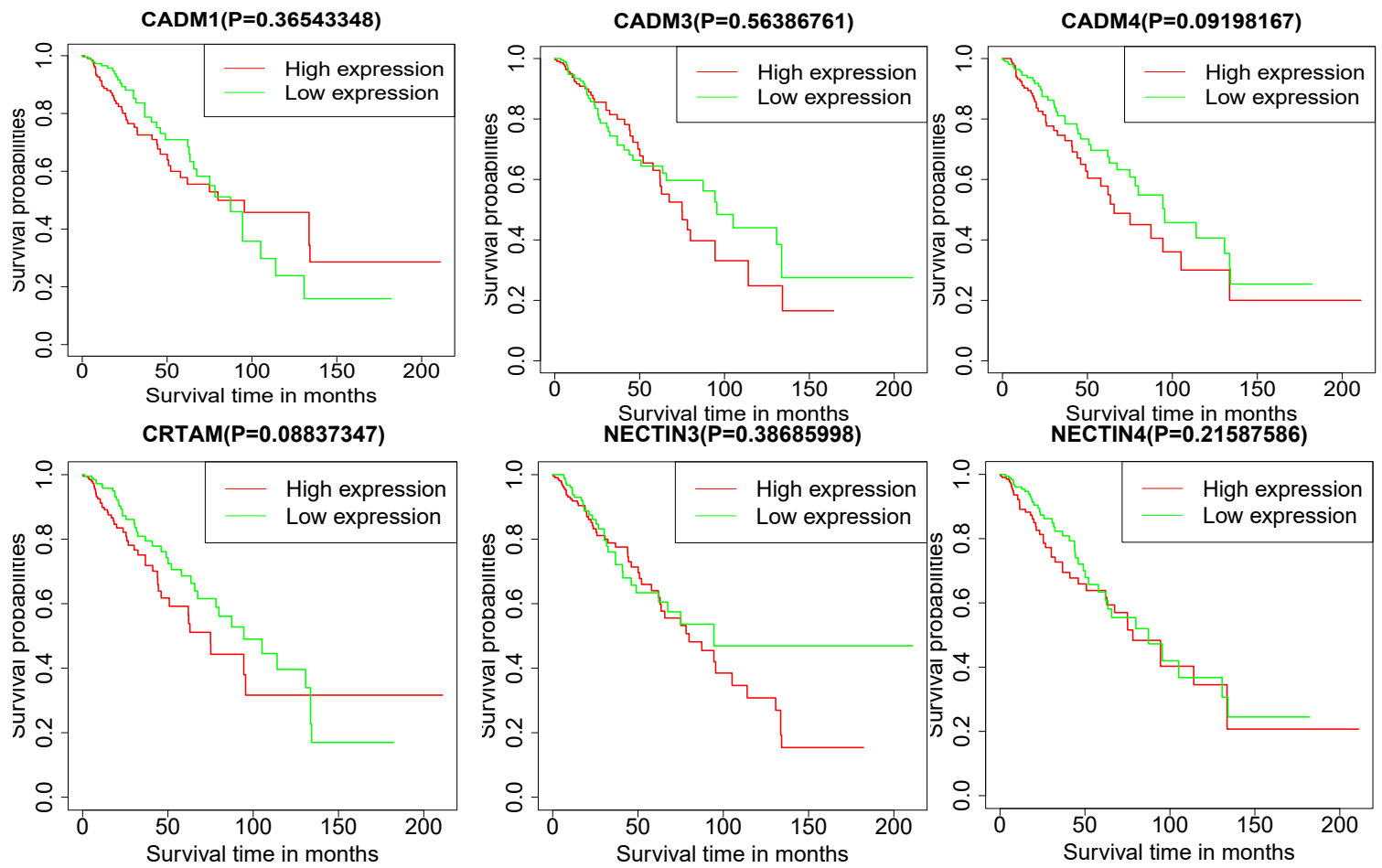

## B CGGA

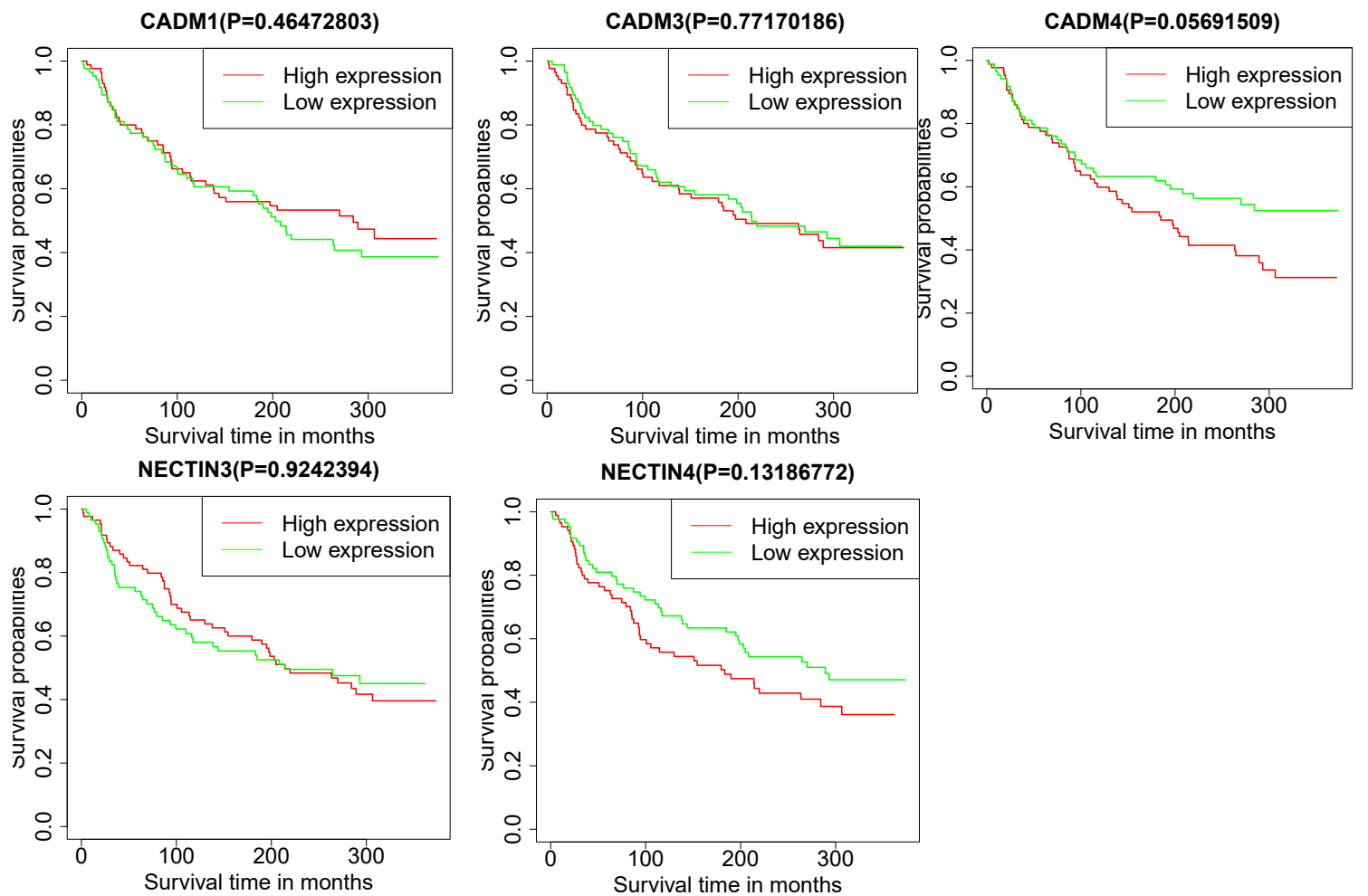

Supplement: Supplementary file 4 [file DataSheet4.PDF]

A

TCGA

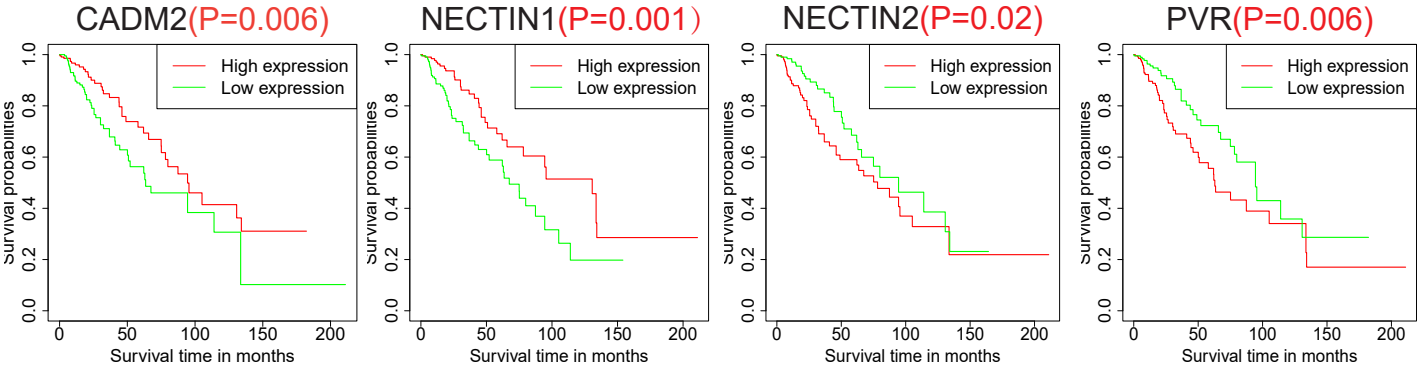

B

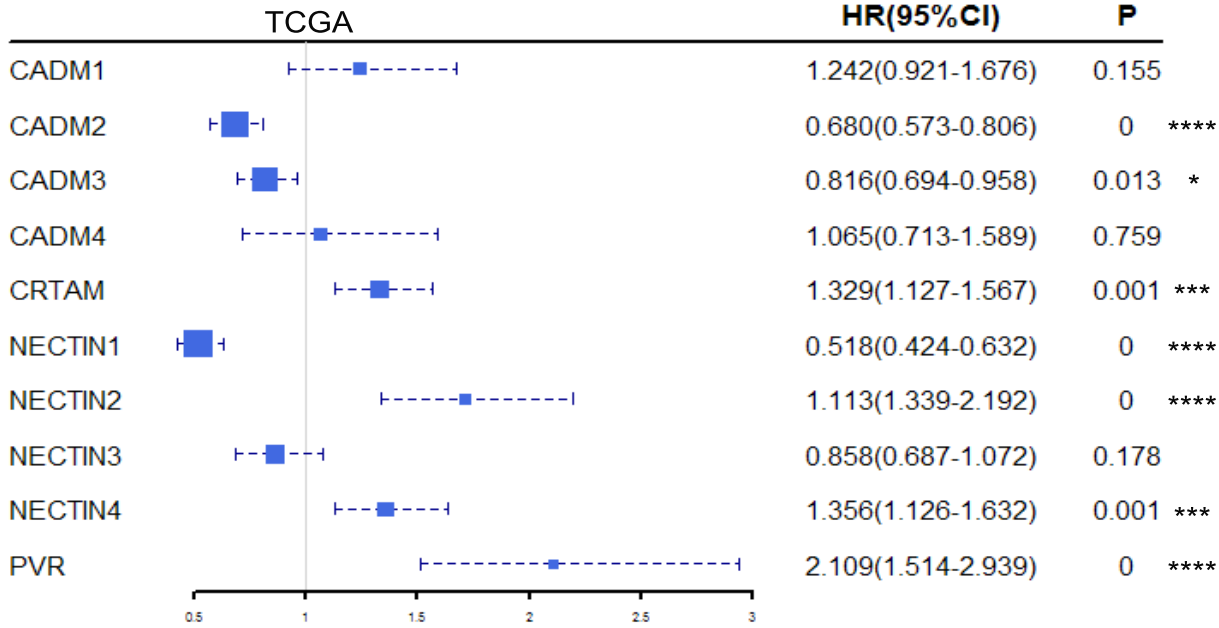

C

CGGA

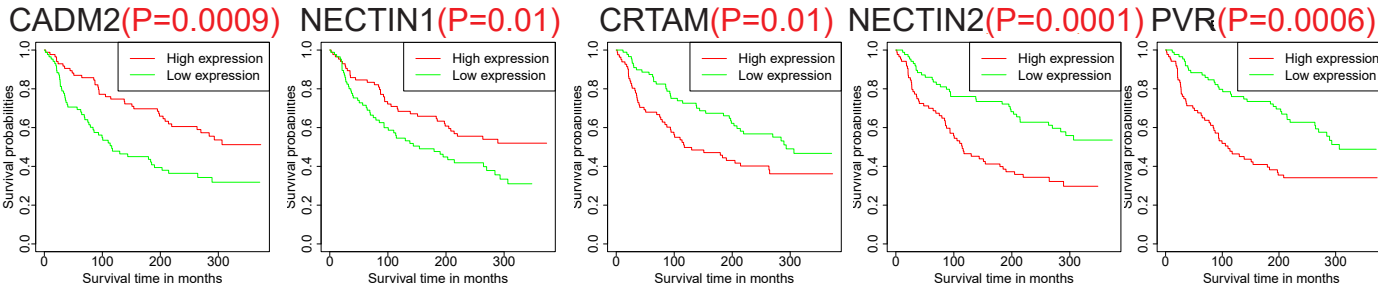

D

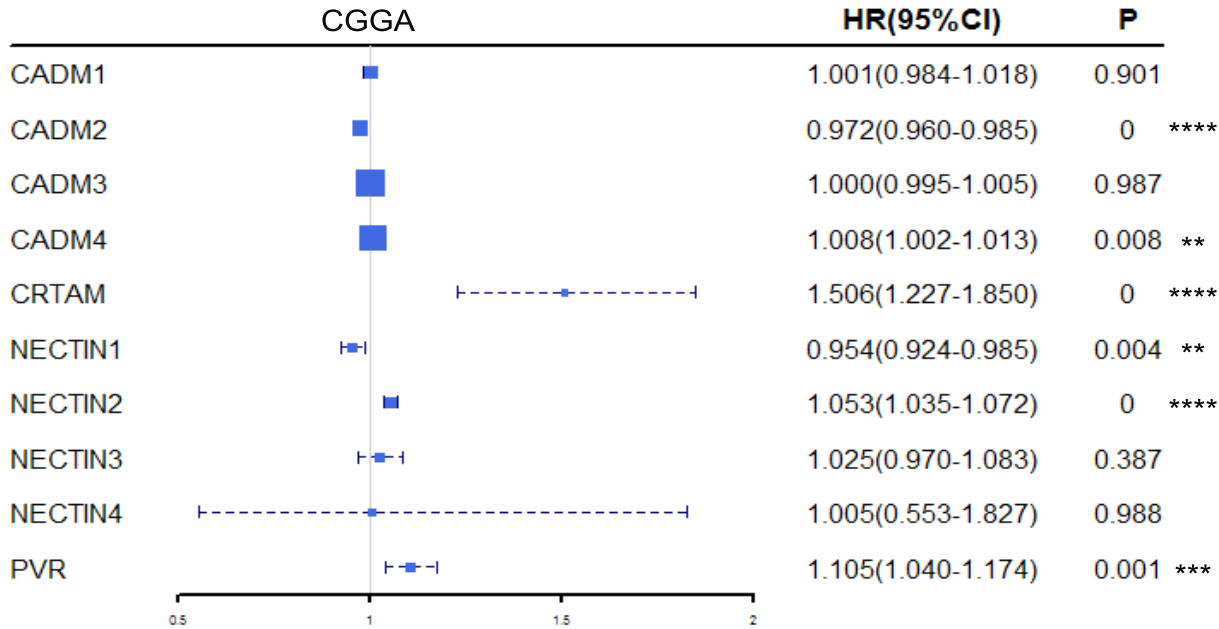

Supplement: Supplementary file 6 [file DataSheet1.ZIP › figure3.pdf]

A

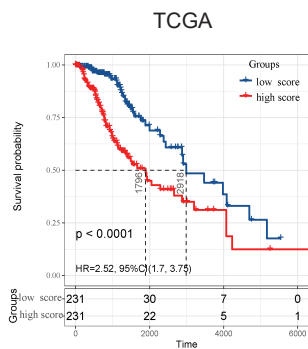

B

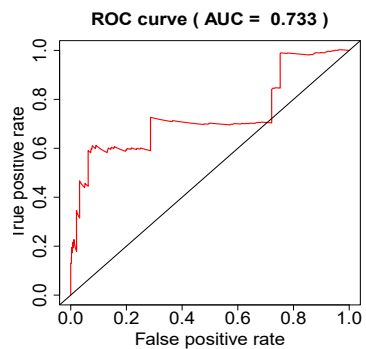

C

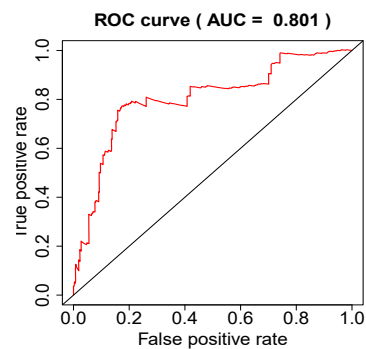

D

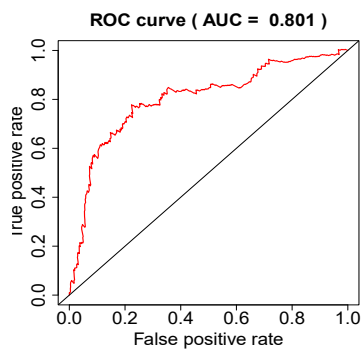

E

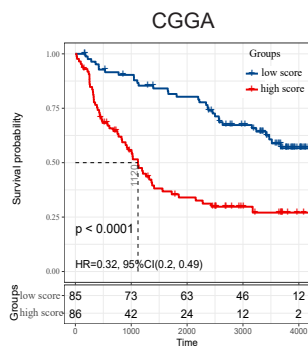

F

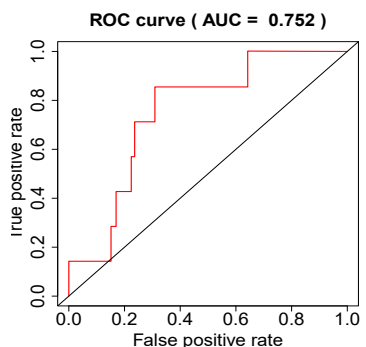

G

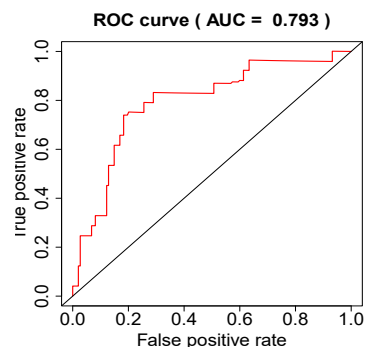

H

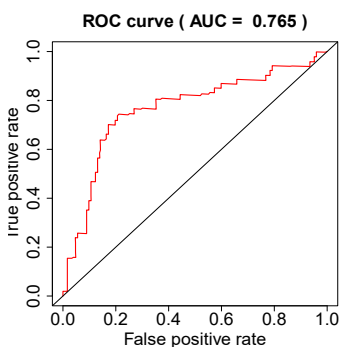

I

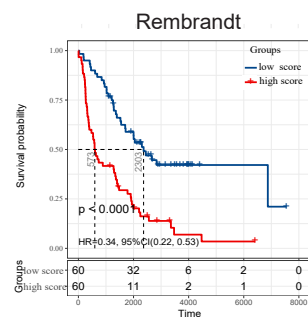

J

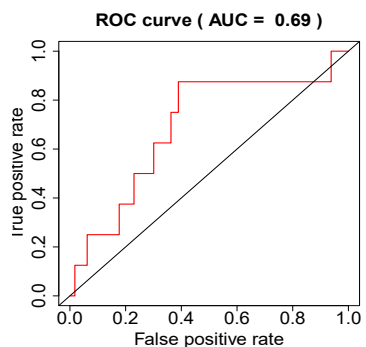

K

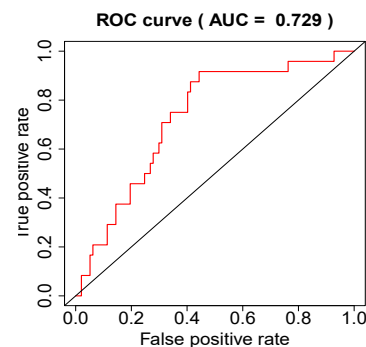

L

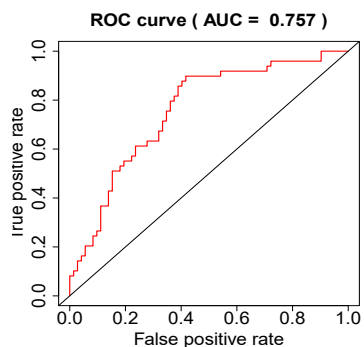

Supplement: Supplementary file 6 [file DataSheet1.ZIP › figure4.pdf]

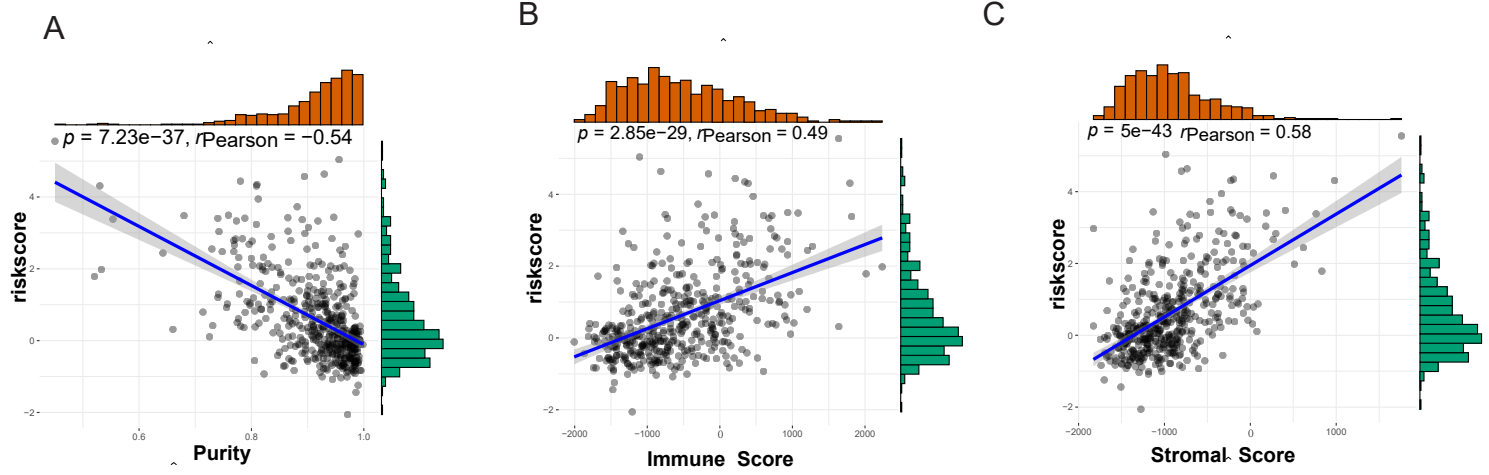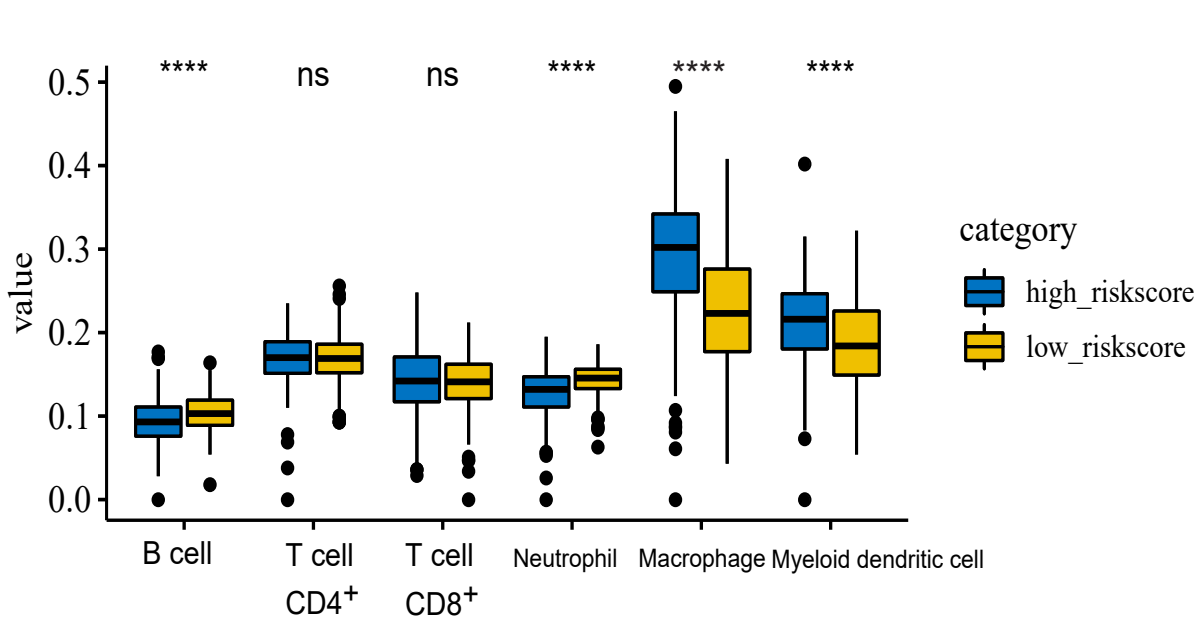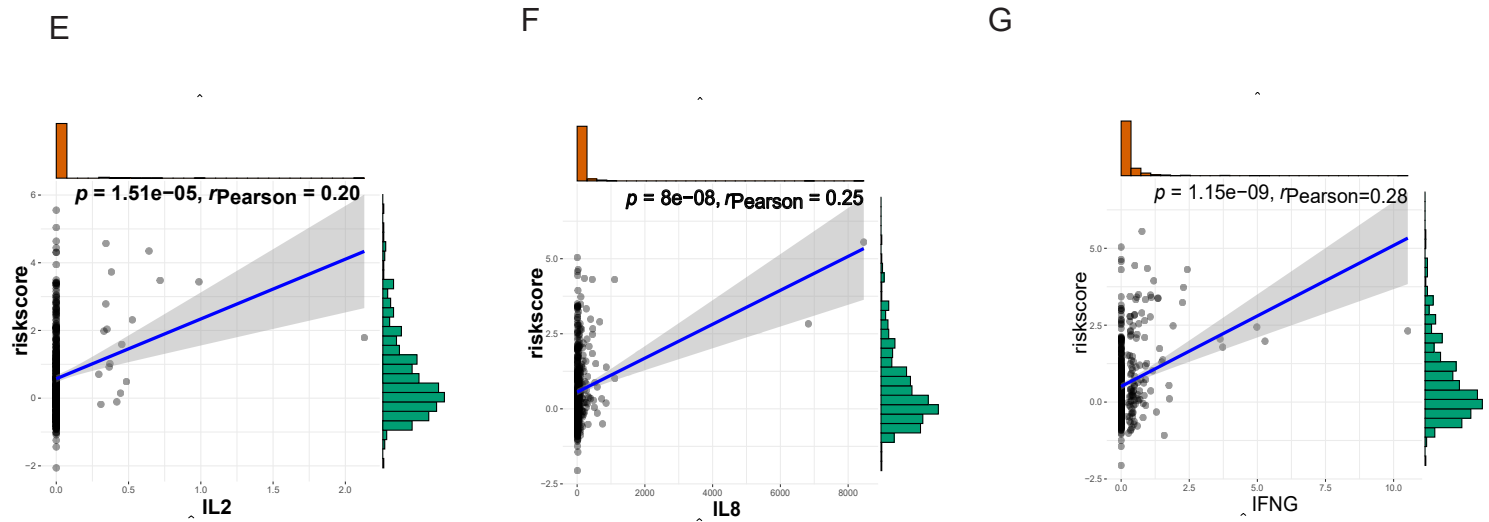

Supplement: Supplementary file 6 [file DataSheet1.ZIP › figure6.pdf]

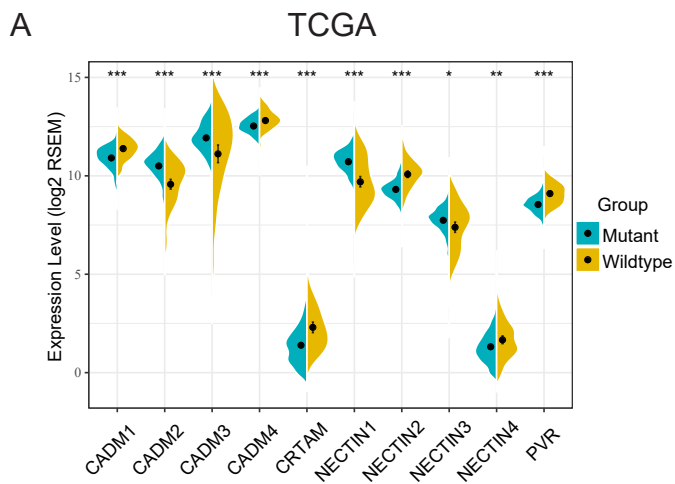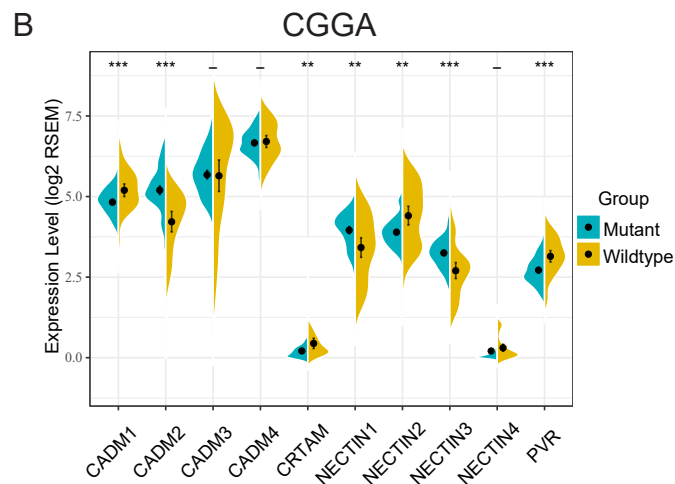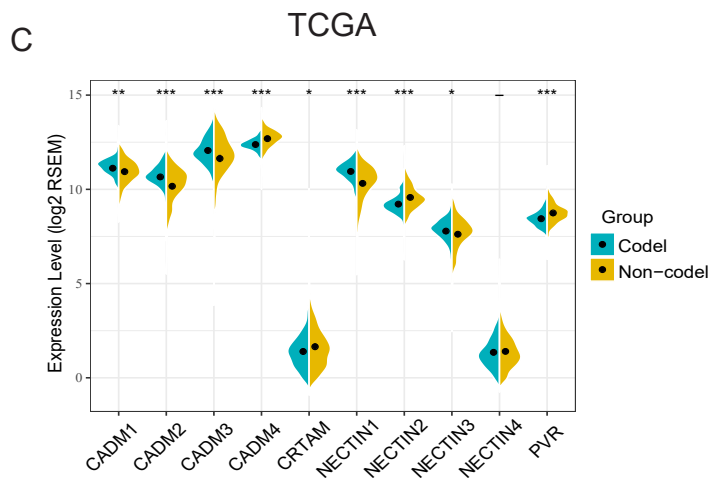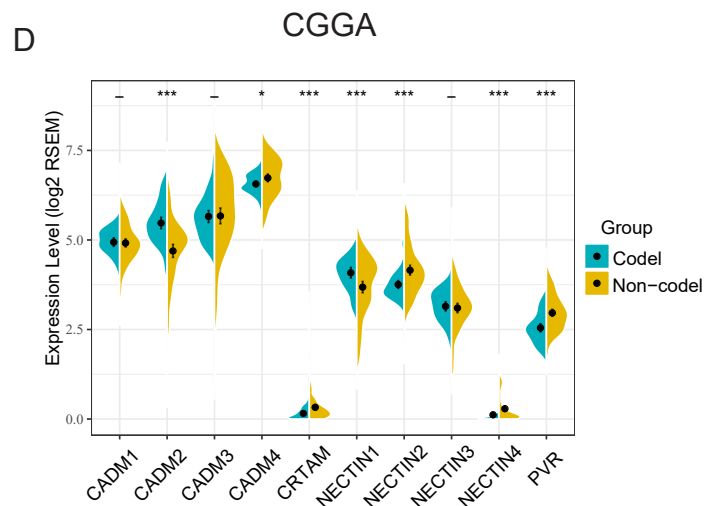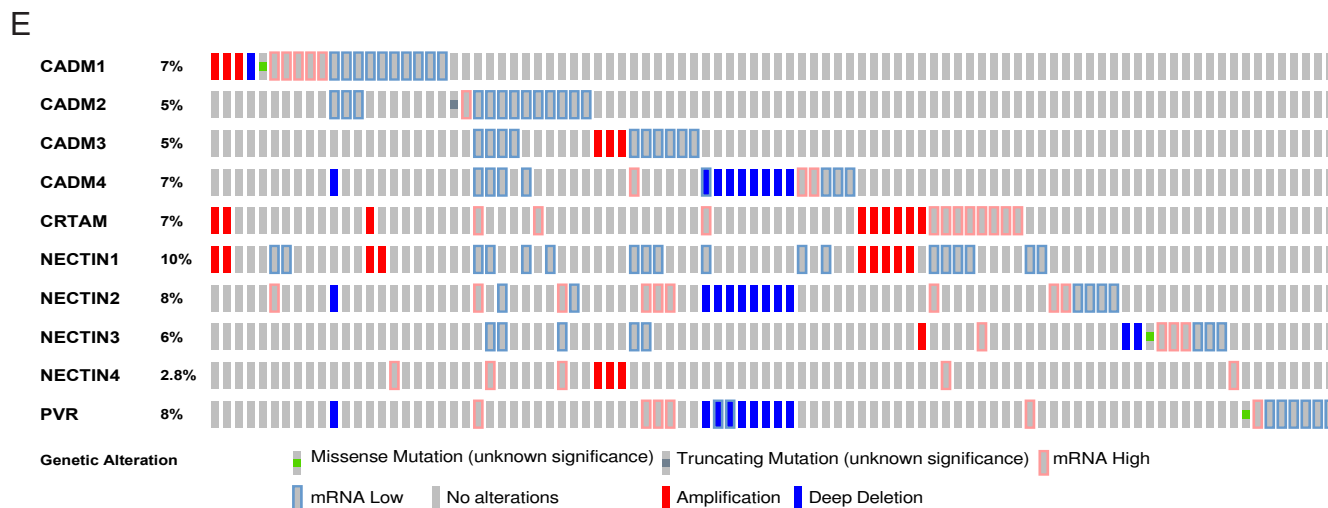

Supplement: Supplementary file 6 [file DataSheet1.ZIP › figure2.pdf]

A

Rembrandt

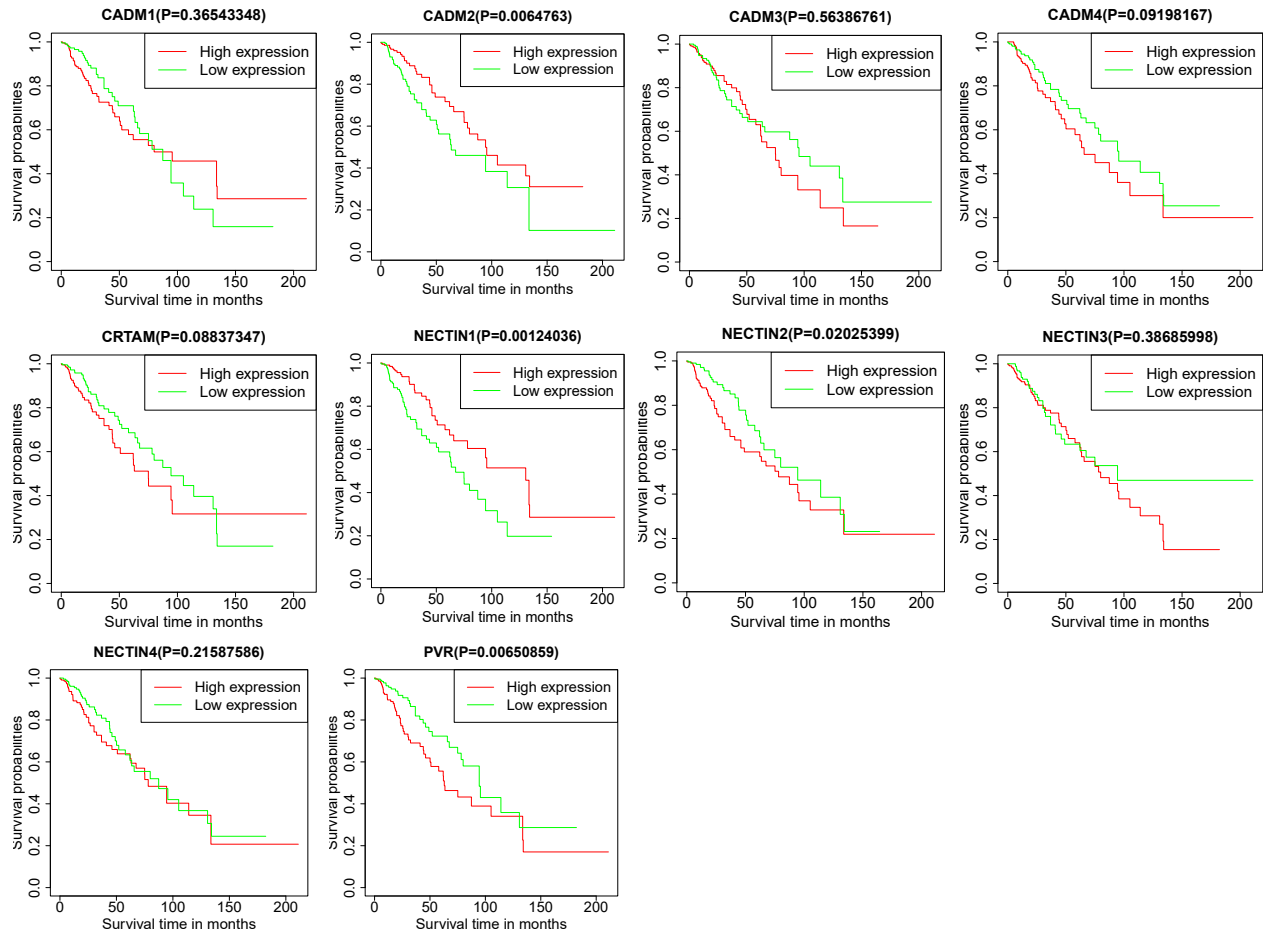

B

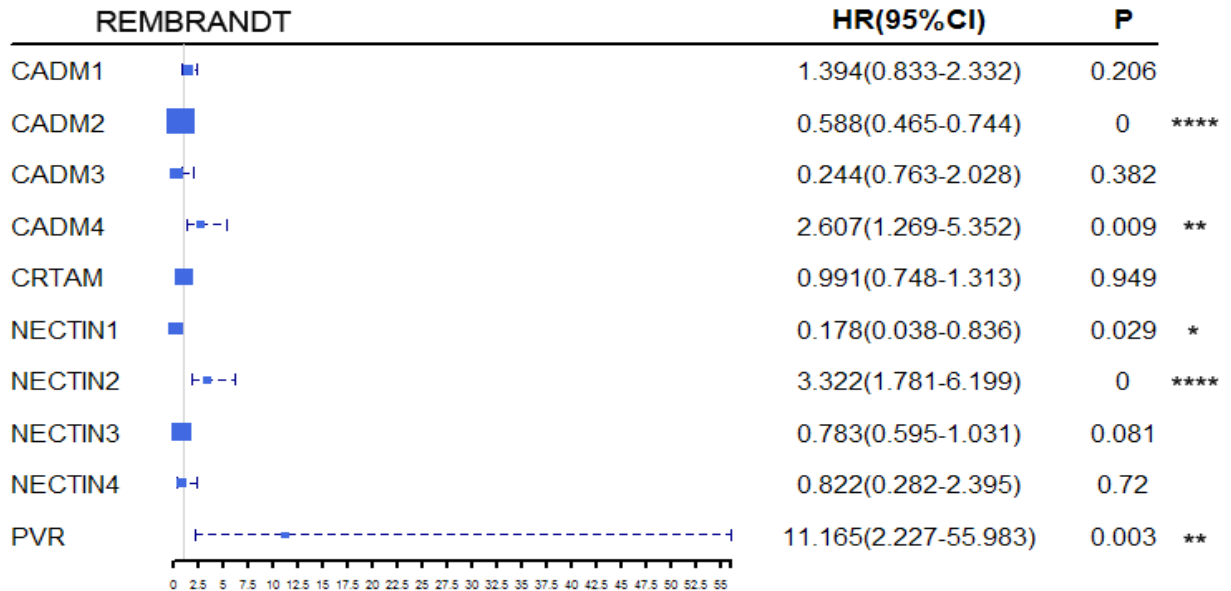

Supplement: Supplementary file 8 [file DataSheet5.PDF]
