# Supplementary material for: The Systematic Landscape of Nectin Family and Nectin-Like Molecules: Functions and Prognostic Value in Low Grade Glioma
Source: Front Genet. 2021 Dec 1;12:718717. doi: 10.3389/fgene.2021.718717 (PMC8672115; doi:10.3389/fgene.2021.718717)
Supplement: Supplementary file 5 [file DataSheet6.PDF]

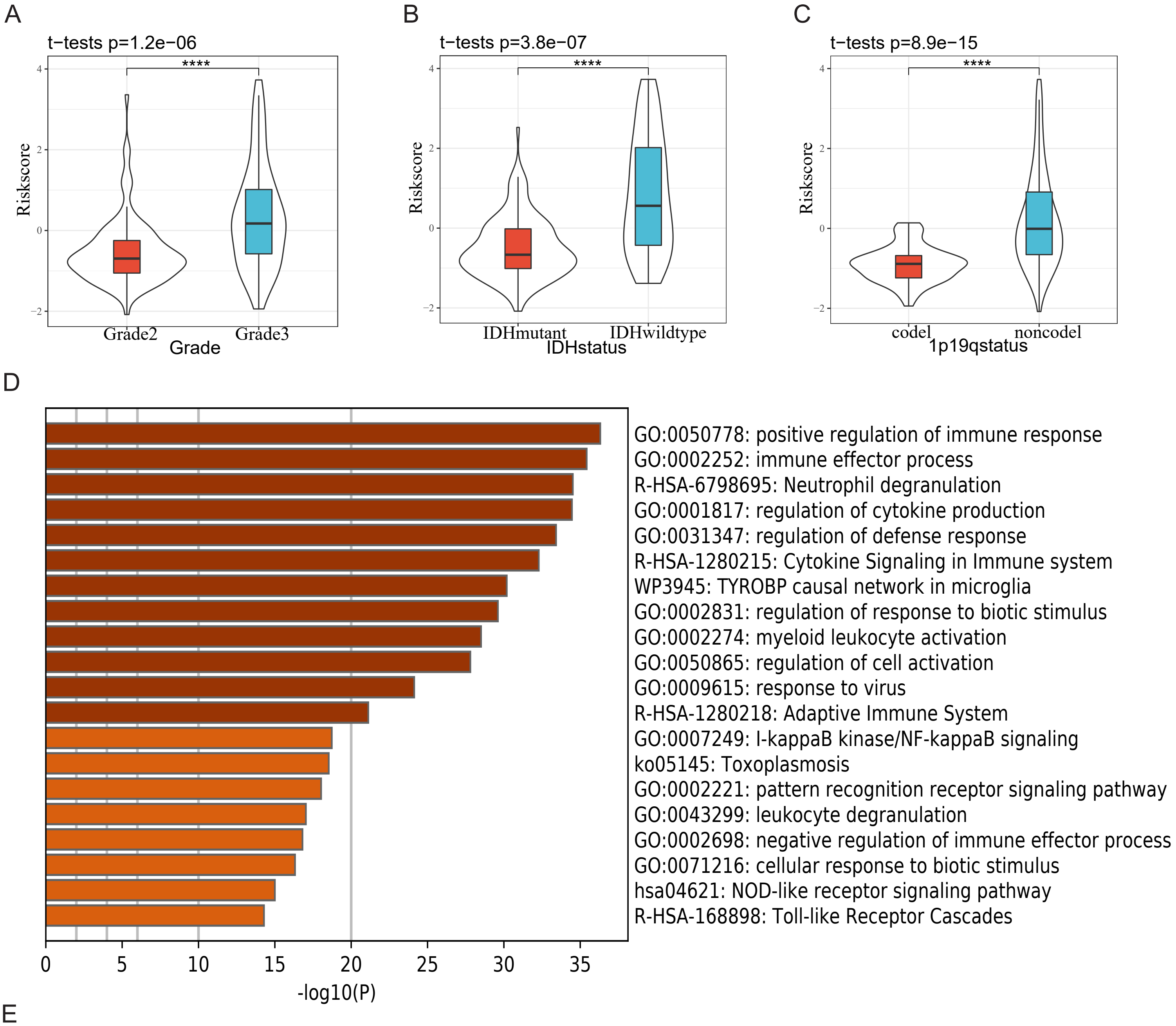

E

NAME	NES	FDR q-val
INTERLEUKIN_2_PRODUCTION	-2.08848	0.32771
POSITIVE_REGULATION_OF_EXTRINSIC_APOPTOTIC_SIGNALING_PATHWAY	-2.06136	0.258285
NEGATIVE_REGULATION_OF_INNATE_IMMUNE_RESPONSE	-2.0547	0.190893
SUPEROXIDE_METABOLIC_PROCESS	-2.04865	0.161165
CELL_REDOX_HOMEOSTASIS	-2.01582	0.225365
INTEGRIN_MEDIATED_SIGNALING_PATHWAY	-2.00131	0.238903
CYTOKINE_METABOLIC_PROCESS	-1.99982	0.210569
T_CELL_PROLIFERATION	-1.99392	0.200866
INTERLEUKIN_8_PRODUCTION	-1.99366	0.179411
RESPONSE_TO_TYPE_I_INTERFERON	-1.99346	0.161691
RESPONSE_TO_VIRUS	-1.99269	0.14886
NEGATIVE_REGULATION_OF_INTERLEUKIN_2_PRODUCTION	-1.98928	0.145198
GLYCOSYL_COMPOUND_CATABOLIC_PROCESS	-1.98631	0.139637
NEGATIVE_REGULATION_OF_LYMPHOCYTE_ACTIVATION	-1.98213	0.137135
NEGATIVE_REGULATION_OF_CELL_ACTIVATION	-1.97385	0.144491
RESPONSE_TO_FUNGUS	-1.97028	0.142688
ANTIGEN_PROCESSING_AND_PRESENTATION_OF_EXOGENOUS_PEPTIDE_ANTIGEN_VIA_MHC_CLASS_I	-1.95615	0.16202
NEGATIVE_REGULATION_OF_T_CELL_PROLIFERATION	-1.95124	0.163441
IMMUNE_EFFECTOR_PROCESS	-1.95065	0.155688
INNATE_IMMUNE_RESPONSE	-1.9484	0.152867
