# Supplementary material for: The Systematic Landscape of Nectin Family and Nectin-Like Molecules: Functions and Prognostic Value in Low Grade Glioma
Source: Front Genet. 2021 Dec 1;12:718717. doi: 10.3389/fgene.2021.718717 (PMC8672115; doi:10.3389/fgene.2021.718717)
Supplement: Supplementary file 6 [file DataSheet1.ZIP › figure1.pdf]

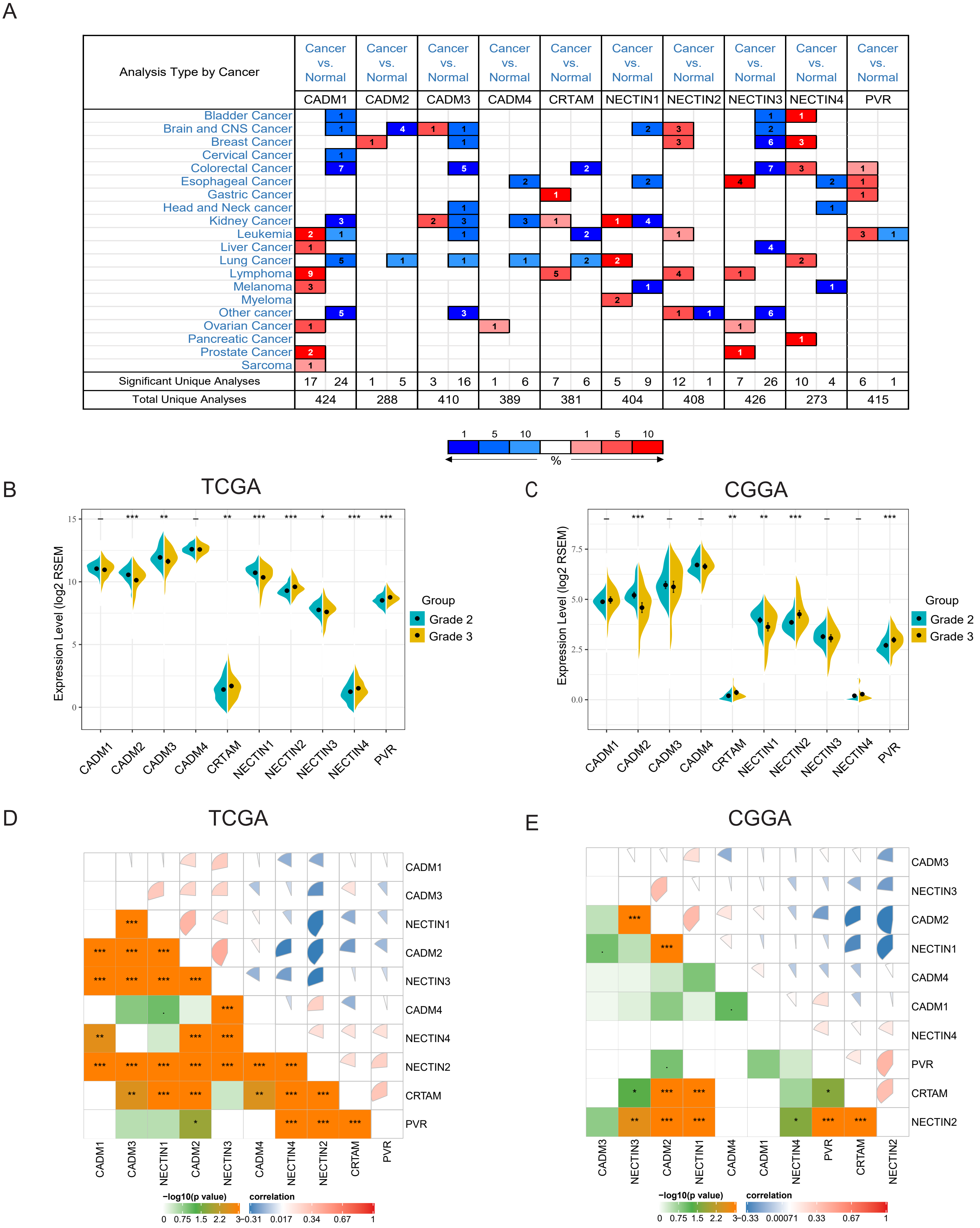
A
Analysis Type by Cancer
Cancer vs. Normal
CADM1
CADM2
CADM3
CADM4
CRTAM
NECTIN1
NECTIN2
NECTIN3
NECTIN4
PVR
Bladder Cancer
Brain and CNS Cancer
Breast Cancer
Cervical Cancer
Colorectal Cancer
Esophageal Cancer
Gastric Cancer
Head and Neck cancer
Kidney Cancer
Leukemia
Liver Cancer
Lung Cancer
Lymphoma
Melanoma
Myeloma
Other cancer
Ovarian Cancer
Pancreatic Cancer
Prostate Cancer
Sarcoma
Significant Unique Analyses
17 24 | 1 5 | 3 16 | 1 6 | 7 6 | 5 9 | 12 1 | 7 26 | 10 4 | 6 1
Total Unique Analyses
424 | 288 | 410 | 389 | 381 | 404 | 408 | 426 | 273 | 415
1 5 10 1 5 10
%
B
TCGA
Expression Level (log2 RSEM)
Group
Grade 2
Grade 3
C
CGGA
Expression Level (log2 RSEM)
Group
Grade 2
Grade 3
D
TCGA
−log10(p value)
correlation
E
CGGA
−log10(p value)
correlation
