# Supplementary material for: The Systematic Landscape of Nectin Family and Nectin-Like Molecules: Functions and Prognostic Value in Low Grade Glioma
Source: Front Genet. 2021 Dec 1;12:718717. doi: 10.3389/fgene.2021.718717 (PMC8672115; doi:10.3389/fgene.2021.718717)
Supplement: Supplementary file 6 [file DataSheet1.ZIP › figure5 .pdf]

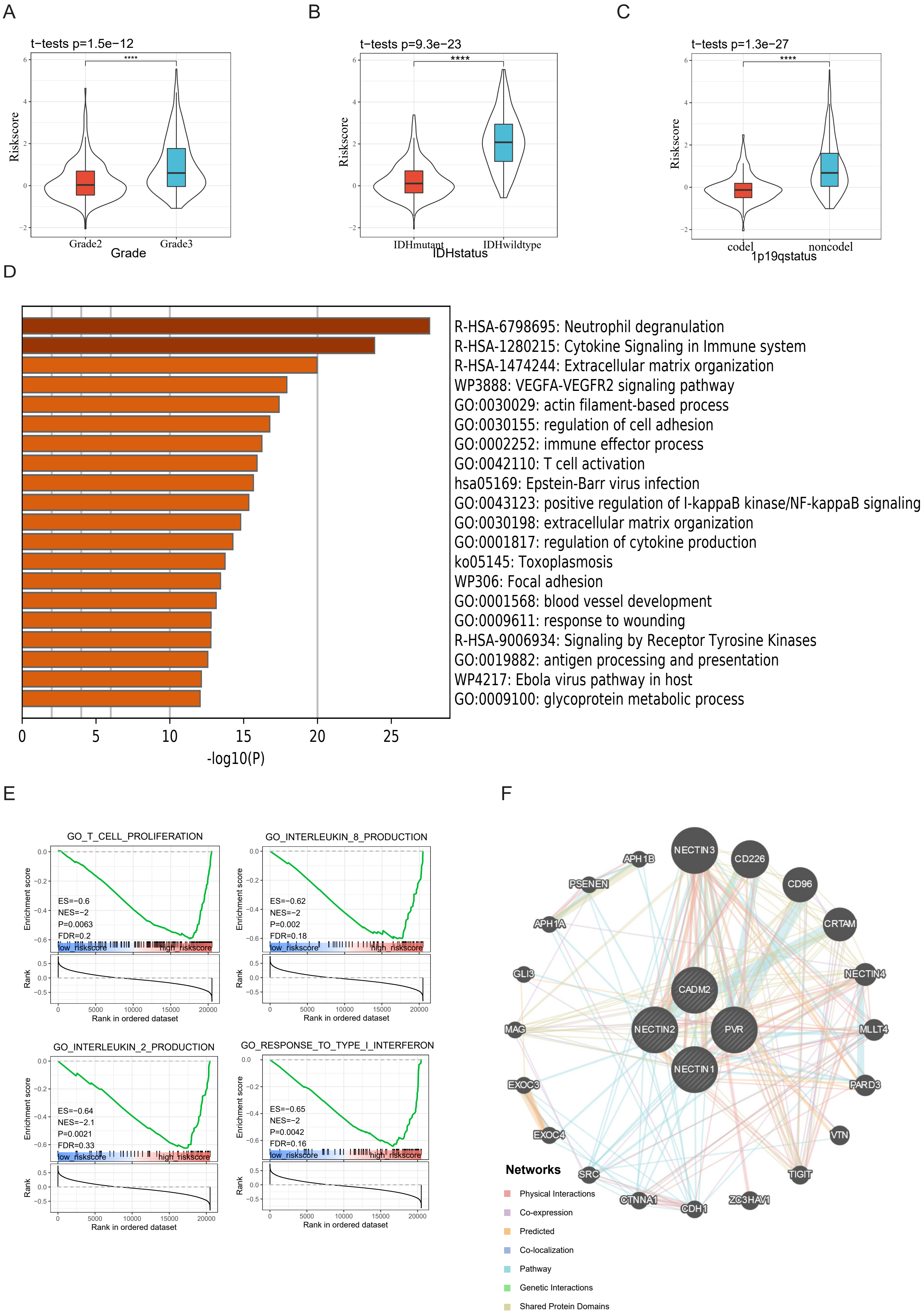
A
B
C
t−tests p=1.5e−12
t−tests p=9.3e−23
t−tests p=1.3e−27
Riskscore
Grade2
Grade3
Grade
IDHmutant
IDHwildtype
IDHstatus
codel
noncodel
1p19qstatus
D
R-HSA-6798695: Neutrophil degranulation
R-HSA-1280215: Cytokine Signaling in Immune system
R-HSA-1474244: Extracellular matrix organization
WP3888: VEGFA-VEGFR2 signaling pathway
GO:0030029: actin filament-based process
GO:0030155: regulation of cell adhesion
GO:0002252: immune effector process
GO:0042110: T cell activation
hsa05169: Epstein-Barr virus infection
GO:0043123: positive regulation of I-kappaB kinase/NF-kappaB signaling
GO:0030198: extracellular matrix organization
GO:0001817: regulation of cytokine production
ko05145: Toxoplasmosis
WP306: Focal adhesion
GO:0001568: blood vessel development
GO:0009611: response to wounding
R-HSA-9006934: Signaling by Receptor Tyrosine Kinases
GO:0019882: antigen processing and presentation
WP4217: Ebola virus pathway in host
GO:0009100: glycoprotein metabolic process
-log10(P)
E
GO_T_CELL_PROLIFERATION
ES=−0.6
NES=−2
P=0.0063
FDR=0.2
GO_INTERLEUKIN_8_PRODUCTION
ES=−0.62
NES=−2
P=0.002
FDR=0.18
GO_INTERLEUKIN_2_PRODUCTION
ES=−0.64
NES=−2.1
P=0.0021
FDR=0.33
GO_RESPONSE_TO_TYPE_I_INTERFERON
ES=−0.65
NES=−2
P=0.0042
FDR=0.16
Enrichment score
Rank
low_riskscore
high_riskscore
Rank in ordered dataset
F
NECTIN3
CD226
CD96
CRTAM
NECTIN4
MLLT4
PARD3
VTN
TIGIT
ZC3HAV1
CDH1
CTNNA1
SRC
EXOC4
EXOC3
MAG
GLI3
APH1A
PSENEN
APH1B
CADM2
NECTIN2
PVR
NECTIN1
Networks
Physical Interactions
Co-expression
Predicted
Co-localization
Pathway
Genetic Interactions
Shared Protein Domains
