# Supplementary material for: The Systematic Landscape of Nectin Family and Nectin-Like Molecules: Functions and Prognostic Value in Low Grade Glioma
Source: Front Genet. 2021 Dec 1;12:718717. doi: 10.3389/fgene.2021.718717 (PMC8672115; doi:10.3389/fgene.2021.718717)
Supplement: Supplementary file 7 [file DataSheet3.PDF]

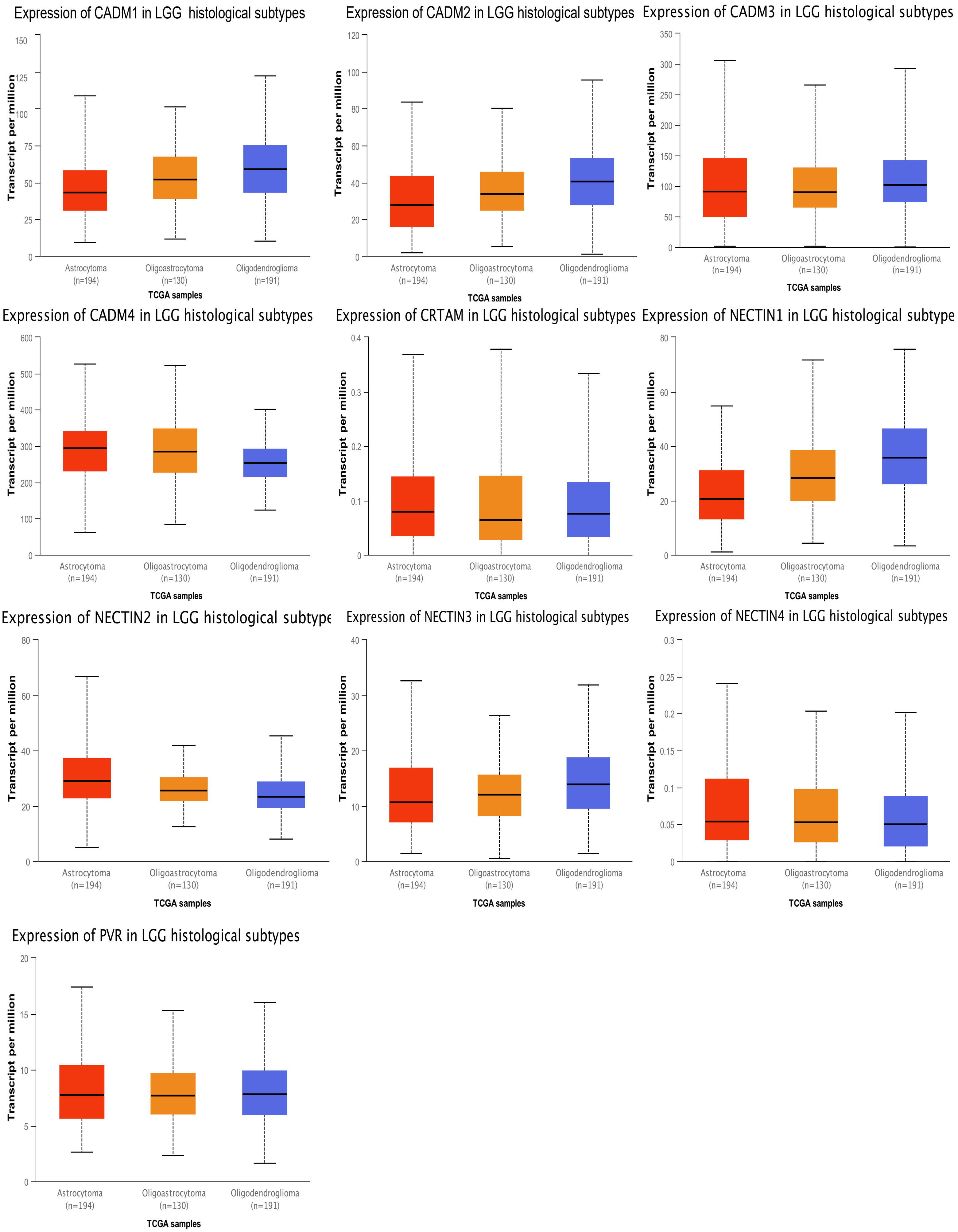
Expression of CADM1 in LGG histological subtypes
Expression of CADM2 in LGG histological subtypes
Expression of CADM3 in LGG histological subtypes
Expression of CADM4 in LGG histological subtypes
Expression of CRTAM in LGG histological subtypes
Expression of NECTIN1 in LGG histological subtype
Expression of NECTIN2 in LGG histological subtype
Expression of NECTIN3 in LGG histological subtypes
Expression of NECTIN4 in LGG histological subtypes
Expression of PVR in LGG histological subtypes
Transcript per million
Astrocytoma (n=194)
Oligoastrocytoma (n=130)
Oligodendroglioma (n=191)
TCGA samples
